# Proteomics and metabolomics profiling reveal panels of circulating diagnostic biomarkers and molecular subtypes in stable COPD

**DOI:** 10.1186/s12931-023-02349-x

**Published:** 2023-03-11

**Authors:** Zili Zhang, Jian Wang, Yuanyuan Li, Fei Liu, Lingdan Chen, Shunping He, Fanjie Lin, Xinguang Wei, Yaowei Fang, Qiongqiong Li, Juntuo Zhou, Wenju Lu

**Affiliations:** 1 State Key Laboratory of Respiratory Diseases, Guangdong Key Laboratory of Vascular Diseases, National Clinical Research Center for Respiratory Diseases, Guangzhou Institute of Respiratory Health, The First Affiliated Hospital of Guangzhou Medical University, Guangzhou, Guangdong China; 2 Department of Respiratory and Critical Care, Shaoguan First People’s Hospital, Shaoguan, Guangdong China; 3 Beijing Advanced Innovation Center for Big Data-Based Precision Medicine, Beihang University, Beijing, 100083 China; 4 Guangzhou Laboratory, Guangzhou, 510005, Guangdong China

**Keywords:** COPD, Proteomics, Metabolomics, Molecular subtypes

## Abstract

**Background:**

Chronic obstructive pulmonary disease (COPD) is a complex and heterogeneous disease with high morbidity and mortality, especially in advanced patients. We aimed to develop multi-omics panels of biomarkers for the diagnosis and explore its molecular subtypes.

**Methods:**

A total of 40 stable patients with advanced COPD and 40 controls were enrolled in the study. Proteomics and metabolomics techniques were applied to identify potential biomarkers. An additional 29 COPD and 31 controls were enrolled for validation of the obtained proteomic signatures. Information on demographic, clinical manifestation, and blood test were collected. The ROC analyses were carried out to evaluate the diagnostic performance, and experimentally validated the final biomarkers on mild-to-moderate COPD. Next, molecular subtyping was performed using proteomics data.

**Results:**

Theophylline, palmitoylethanolamide, hypoxanthine, and cadherin 5 (CDH5) could effectively diagnose advanced COPD with high accuracy (auROC = 0.98, sensitivity of 0.94, and specificity of 0.95). The performance of the diagnostic panel was superior to that of other single/combined results and blood tests. Proteome based stratification of COPD revealed three subtypes (I–III) related to different clinical outcomes and molecular feature: simplex COPD, COPD co-existing with bronchiectasis, and COPD largely co-existing with metabolic syndrome, respectively. Two discriminant models were established using the auROC of 0.96 (Principal Component Analysis, PCA) and 0.95 (the combination of RRM1 + SUPV3L1 + KRT78) in differentiating COPD and COPD with co-morbidities. Theophylline and CDH5 were exclusively elevated in advanced COPD but not in its mild form.

**Conclusions:**

This integrative multi-omics analysis provides a more comprehensive understanding of the molecular landscape of advanced COPD, which may suggest molecular targets for specialized therapy.

**Supplementary Information:**

The online version contains supplementary material available at 10.1186/s12931-023-02349-x.

## Introduction

Chronic obstructive pulmonary disease (COPD) is a progressive lung disease characterized by chronic inflammation, airway obstruction, and destruction of the parenchyma. It is the fourth leading cause of death globally, and is projected to be the third leading cause of mortality by 2030 [20]. Current therapies for patients with advanced COPD mainly treat symptoms such as chronic cough and excessive sputum production, as well as prevent disease progression. However, 46–91% of adults still suffer from persistent and disabling breathlessness at rest and on minimal exertion [25]. To date, no therapy has been developed for reducing disease progression and lower mortality rates. Therefore, additional approaches for accurate diagnoses of advanced COPD are urgently needed.

In 2011, the Society for Qualification of Biomarkers for COPD was established to accelerate research and development of biomarkers. To date, however, only a handful of biomarkers associated with COPD have been discovered [21]. Integrated multi-omics data analysis can provide insights into the pathological mechanisms of COPD. Analysis of the proteome can provide studying disease-related mechanisms and diagnostic biomarkers, which reveals disease phenotype [21]. Compared to traditional proteomic techniques, TMT-LC–MS/MS is a more comprehensive and efficient method for capturing and quantification of proteins, with a smaller sample requirement without offset. In addition, the metabolome, which is defined as the total collection of small molecular metabolites present in a given type of cell or organism, is the final downstream product of metabolism. Particularly, it provides an exact reflection of the current metabolic status of the organic body. To date, some progress has been made in the fields of functional proteomics and metabolomics. For example, researchers have applied proteomic approaches to identify novel biomarkers, such as plasma sRAGE for detecting presence and progression of emphysema [33], whereas others have adopted metabolomics approaches to identify potential disease severity markers or therapeutic candidates such as purines [8], sphingolipids [2], and glycerol phospholipids [4]. However, no discovery-based approach has yet resulted in validated clinical biomarkers. Although findings from these omics-centric studies have added to the existing knowledge base, there are several gaps that are yet to be filled. We hypothesize that integrating contemporary proteomics and metabolomics approaches can effectively evaluate metabolic pathways and diagnostic biomarkers in advanced COPD. Moreover, most of the previous multi-omics studies have focused on patients derived from European, American, and African populations [24]. Therefore, it is important to systematically analyze the metabolic and proteomic profile of Chinese patient cohorts to generate new insights for this region.

Patients with COPD are often predisposed to various co-morbidities, such as cardiovascular disease, metabolic syndrome, and bronchiectasis [15, 20, 22]. Additionally, smoking is a risk factor for such co-morbidities, with previous evidence showing that some smokers develop a predominately emphysema phenotype, characterized by alveolar damage, while others developing predominantly airway disease. Evidence from other studies has shown that proteases, inflammation, oxidative stress, immune defects, and infections play a role in the development and progression of COPD [25]. Since COPD is a heterogeneous disease, grading the severity and identifying phenotypes according to the concomitant diseases (i.e., subpopulations of subjects with similar disease characteristics) can expand our understanding of the biological mechanisms underlying the disease’s development and progression. This will facilitate accurate diagnoses of the disease. Particularly, lowering mortality rates in patients with advanced COPD relies on early and accurate diagnosis and differentiation of different subtypes using simple and objective diagnostic assessments. The heterogeneity of COPD also exists at the molecular level, and thus molecular sub-phenotyping is the first and crucial step in the identification and classification of these subgroups. Previous studies have shown that omics approaches, based on appropriate sample sizes, can not only efficiently reveal heterogeneity of these subtypes but also facilitate diagnosis and reveal the exact mechanisms underlying COPD subgroups [22, 24]. Proteomics techniques, based on mass spectrometry, have shown strong power in detecting disease phenotypes.

In this study, we hypothesized that changes in proteomic and metabolic profiles of patients with stable COPD would produce a unique pattern of molecules compared to those without COPD, and that these molecular profiles would change with disease complications. Therefore, we first performed quantitative shotgun proteomic analyses to investigate COPD-related proteins molecular portrait and reveal COPD-related functional modulation. Next, we applied a targeted proteomics approach to validate specific members of dysregulated proteins in another independent sample set. In addition, untargeted metabolomics was performed using the same participants as the proteome. Our findings not only reveal the profiles of COPD biomarkers and molecular subtypes, but also provide data that will guide future studies seeking to develop tools for clinical application.

## Methods

### Biospecimen collection and clinical data

The previously published design of GIRD COPD Biobank (Clinical trial www.chictr.org.cn, number ChiCTR-CCC-12002950) was adopted in this study [19]. The GIRD COPD Biobank collection, which was established in 2010, comprises specimens from patients and controls for research purposes. All patients were diagnosed with COPD through pulmonary function testing and clinical symptoms. The enrolled patients and controls were aged between 40 and 80 years, and were permanent residents (lived in Guangzhou for years). Permission to access medical records was sought from each individual, after which his/her management information was retrieved before and after hospitalization. The participants were also requested to provide blood samples and other materials for research purposes. The cross-sectional analysis presented in this work is based on clinical and biomarker data obtained at baseline.

Inclusion criteria of patients were as follows: male subjects aged 55–75 years, had a Global Initiative for Obstructive Lung Disease (GOLD) stage 1–4, and were current or ex-smokers with a smoking history of greater than or equal to 10 packyears, as well the patients were all stable COPD without therapies including steroid, theophylline, antibiotics etc. at least 1 week before joining the group. The exclusion criteria were: patients suffering from lung disease except extensive bronchiectasis, such as cystic fibrosis, and pulmonary fibrosis; with other inflammatory diseases, or reported COPD exacerbation within 4 weeks of enrollment. Participants were assigned to the control group if they had normal spirometry, cancer-free without suffering from any lung disease, aged between 40 and 80 years, current or ex-smokers, and were permanent residents of Guangzhou (lived in Guangzhou for several years).

**Fig. 1:**
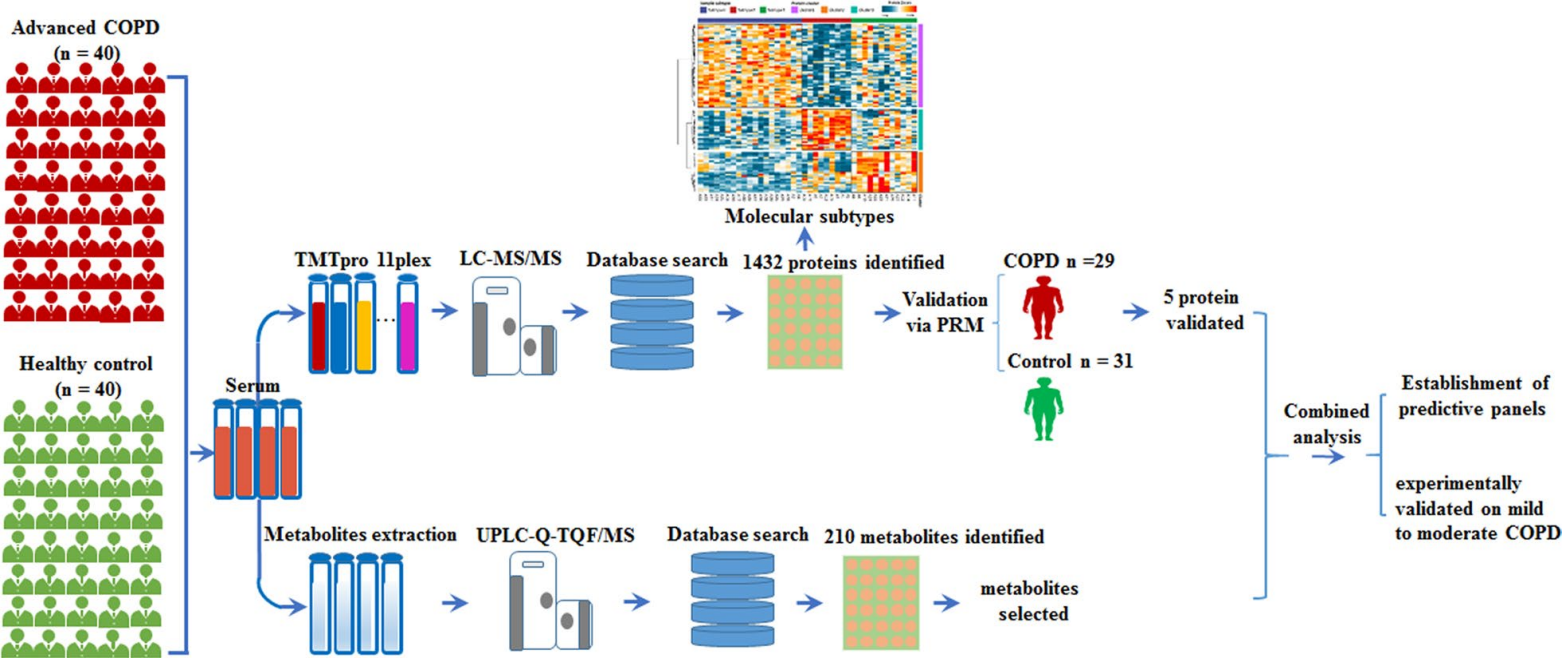
Overview of the experimental design and the number of samples for proteomics, metabolomics, and protein validation. 40 stable advanced COPD along side 40 controls were recruited, then applied proteomics and metabolomics techniques to detect potential biomarkers. An additional 30 COPD vs 30 controls was used to validate the resultant proteomic signatures. Molecular subtyping performed using proteomics data. Receiver operating characteristics (ROC) analyses used to evaluate predictive capability of the biomarkers, and then experimentally validated the predictions on mild-to-moderate COPD

Finally, 70 advanced COPD patients and 70 healthy controls were recruited for multi-omics study. Additional 10 mild-to-moderate COPD and 11 healthy controls were enrolled for protein biomarker validation. For the validation of final metabolites, the results of 46 mild-to-moderate COPD and 48 healthy controls were obtained with the help of professor Zhou [36]. For proteomics analysis, subjects were divided into a discovery (comprising 40 COPD patents and 40 controls) and validation (30 COPD patients and 30 controls) groups. Metabolomics analyses were only performed on the discovery cohort. Blood samples were obtained from all participants before breakfast, and immediately processed according to a previously published standard protocol [19]. Briefly, blood was collected into serum separating tubes (SST, Vacutainer SST II Tube 8.5 mL, #368972; BD), manually inverted 10 times, then centrifuged for 10 min at 1300×*g*. Serum samples were aliquoted and stored at − 80 °C until proteomics analysis. Peripheral Blood Mononuclear cells (PBMCs) were isolated using lymphocyte separation medium as described previously [34]. The study design is shown in Fig. 1.

Furthermore, we collected each patient’s demographic, clinical manifestation, anthropometric information, individual and family disease history, and other co-morbidities. Moreover, information on other parameters, such as indoor living and working environment, dietary habits and smoking habits was collected. Family history of cancer was defined as any self-reported cancer in his/her first-degree relatives, such as parents, siblings, or children. A summary of the characteristics of all COPD patients and control subjects with complete data is presented in Table 1 in discovery stage. Each patient voluntarily provided a written informed consent prior to inclusion in the study and data collection. The study was approved by the Institutional Review Board of Guangzhou Medical University, Ethics Committee of the First Affiliated Hospital (approval number: GZMC 2009-08-1336), and was conducted in accordance with the principles of the Declaration of Helsinki.

**Table 1:** Characteristics of proteomics cohorts in discovery stage and in validation stage

	Discovery stage		*P*^a^	Validation stage		*P*^a^
	COPD	Controls		COPD	Controls	
N	40	40		29	31	
Male, %	100	100	1.0	100	100	1.0
Smoking, %	100	100	1.0	100	100	1.0
Packyears ≥ 30, %	100	100	1.0	100	100	1.0
Age (years), mean (SD)	64.6 (11.7)	63.7 (5.3)	0.656			
Height (cm), mean (SD)	165.0 (5.2)	166.0 (5.9)	0.824	164 (4.9)	169 (5.9)	**< 0.001**
Weight (kg), mean (SD)	63.1 (12.5)	65.7 (8.2)	0.266	60.60 (9.3)	69.10 (9.4)	**0.001**
BMI (kg/m^2^), mean (SD)	23.8 (2.9)	23.1 (4.6)		22.40 (3.2)	23.90 (2.5)	0.054
Fan in kitchen, %	93.2	92.7	0.475	92.3	93.6	1.000
Good room ventilation, %	38.6	63.4	**0.022**	62.1	51.6	0.414
Often preserved food, %	18.8	7.1	0.106	10.3	3.2	0.346
Often cook, %	25.0	29.3	0.658	17.2	35.5	0.110
Comorbidity, %						
CRD	29.8	12.2	0.045	34.6	3.2	**0.002**
Hypertension	42.6	31.7	0.294	34.6	22.6	0.314
Diabetes	12.8	7.5	0.498	0	10	–
Heart diseases	40.4	12.5	**0.004**	19.2	6.7	0.231
Stroke	4.3	0	–	3.8	0	–
Family history, %						
Cancer	6.3	9.5	0.563	23.1	22.6	0.965
RD without COPD	19.1	15.8	0.759	7.1	3.2	–
Severe COPD, %	100	0	–	100	0	–
Pulmonary function, mean (SD)						
pre_FVC_%Pred	74.9 (28.1)	98.0 (15.0)	**< 0.001**	78.5 (16.3)	94.3 (10.4)	**< 0.001**
pre_FEV1_%Pred	59.7 (32.1)	88.4 (26.1)	**< 0.001**	47.1 (14.3)	94.5 (9.6)	**< 0.001**
pre_FEV1/FVC_%Pred	62.7 (15.9)	71.3 (15.5)	**0.036**	57.4 (15.8)	96.8 (9.7)	**< 0.001**
post_FVC_%Pred	74.6 (24.6)	99.8 (12.5)	**< 0.001**	88.3 (14.6)	104 (13.0)	0.061
post_FEV1_%Pred	57.1 (28.6)	90.8 (25.4)	**< 0.001**	53.8 (15.8)	101 (10.4)	**< 0.001**
post_FEV1/FVC_%Pred	60.7 (15.2)	70.9 (15.4)	**0.021**	57.7 (15.4)	76.9 (6.3)	**0.025**

Bold values indicate significant differences

*CRD* chronic respiratory disease, *severe COPD* Global Initiative for Obstructive Lung Disease stage 3–4

^a^*P-*values for a two-sided χ^2^ test or t-test. Data are median (P_25_–P_75_), n (%)

### Proteomics analysis

#### Protein extraction and trypsin digestion of identified proteomics

Cellular debris were first removed from serum samples via a 10-min centrifugation at 12,000×*g* at 4 °C, and the supernatant transferred to a new centrifuge tube. The top 12 high abundance proteins were then removed by Pierce™ Top 12 Abundant Protein Depletion Spin Columns Kit (Thermo Fisher), and the protein concentration determined using the BCA kit according to the manufacturer’s instructions. For digestion, the protein solution was reduced by treating it with 5 mM dithiothreitol for 30 min at 56 °C, then alkylated with 11 mM iodoacetamide for 15 min at room temperature in darkness. Next, the prote in sample was diluted by adding 100 mM TEAB to urea concentration less than 2 M. Trypsin, at 1:50 trypsin-to-protein mass ratio, was added for the first digestion overnight, followed by 1:100 trypsin-to-protein for a second 4 h-digestion. After tryps indigestion, peptide was desalted using Strata X C18 SPE columns (Phenomenex), vacuum-dried, then reconstituted in 0.5 M TEAB and labeled for TMT pro11 plexkit according to the manufacturer’s protocol.

#### Liquid chromatography–mass spectrometry (LC–MS/MS)

The tryptic peptides were fractionated into fractions by high pH reverse-phase HPLC using Thermo Betasil C18 column (5 μm particles, 10 mm ID, 250 mm length). The peptides were first separated with a gradient of 8% to 32% acetonitrile (pH 9.0) over 60 min into 60 fractions. Then, the peptides were combined into 6 fractions and dried by vacuum centrifuging. An electrospray, at a voltage of 2.0 kV, was applied with a m/z scan range of 350 to 1800 for full scan, while intact peptides were detected in the Orbitrap at a resolution of 70,000. The peptides were then selected for MS/MS using NCE setting as 28, while the fragments were detected in the Orbitrap at a resolution of 17,500. A data-dependent procedure, which alternated between one MS scan followed by 20 MS/MS scans with 15.0 s dynamic exclusion, was also applied. Automatic gain control (AGC) was set at 5E4, while the fixed first mass was set at 100 m/z.

#### Analysis of proteomics data

Differentially expressed proteins (DEPs) were identified using the empirical Bayesian algorithm implemented in the limma package in R software. Up-regulated and down-regulated proteins were defined by a fold change of ≥ 1.2 or ≤ 0.83 and a *P*-value < 0.05. We performed Gene Ontology (GO) annotation of the proteome, using the UniProt-GOA database (http://www.ebi.ac.uk/GOA), then identified enriched pathways using the Kyoto Encyclopedia of Genes and Genomes (KEGG) analysis. Next, we used the “heatmap.2” function in “ggplot” package in R to perform hierarchical clustering and visualization of the DEPs. We used the GO terms to classify the proteins into three categories, namely biological process, cellular components, and molecular function. Accession numbers for all DEPs or their sequences were searched against the STRING database version 10.1 for protein–protein interactions (PPI). The STRING algorithm uses a metric called “confidence score” to define interaction confidence. Thus, we fetched all interactions with a confidence score of ≥ 0.7 (high confidence). The resulting interaction was among proteins was visualized using the “networkD3” package in R.

Principal Component Analysis (PCA) is a data reduction technique used to convert a large set of variables into a smaller set which still contains most of the original information. Principal components were extracted as a linear combination of the variables. This variance was then removed and a second linear combination was built, which iteratively explains the maximum proportion of the whole information. This is called the principal axis method, which leads to orthogonal (uncorrelated) factors. Furthermore, it involves the computation of eigenvalues and eigenvectors of covariance matrices. These eigenvectors were sorted in the descending order of their eigenvalues, followed by the actual data [13].

#### Validation using targeted proteomics analysis

For validation, 30 μL of serum, collected as earlier described, was treated with Pierce™ Top 12 Abundant Protein Depletion Spin Columns Kit (Thermo Fisher) according to the manufacturer’s instructions. The mixture was digested with trypsin in a similar fashion to the discovery study. All samples were analyzed via LC–MS, operated under the parallel-reaction monitoring (PRM) acquisition scheme. PRM data were analyzed using Skyline (v.3.6) to identify transitions and peak area integration, while protein intensities were Log_2_ transformed. Proteins with missing values, in more than 60% samples, were excluded, while the remaining missing values were considered to be low abundance due to limited MS sensitivity. Therefore, we replaced them using random numbers drawn from a normal distribution with a mean value 1.8× lower and a standard deviation 0.3× of the original data.

### Metabolomics analyses

#### Untargeted metabolomics analysis using UPLC-Q-TOF/MS

Metabolic profiling of serum samples was performed on an Agilent 1290 Infinity LC system (Agilent Technologies, Santa-Clara, California, USA), coupled with an AB SCIEX Triple TOF 6600 System (AB SCIEX, Framingham, MA, USA). Chromatographic separation and aqueous phase of extracts used for both positive and negative models, was implemented on ACQUITY HSS T3 1.8 μm (2.1 × 100 mm) columns with a temperature of 25 °C. The mobile phases, comprising 0.1% formic acid in water (A) and 0.1% formic acid in acetonitrile (B), were used in the positive ionization mode, while 0.5 mM ammonium fluoride in water (C) and acetonitrile (D) were used in negative ionization mode. In the positive (negative) model, the elution gradient initially started with 1% B (D) for 1 min, linearly increased to 100% B (D) at 8 min, where it was maintained for 2 min, then returned to 1% B (D) for about 2 min of equilibrium. Delivery was achieved at a flow rate of 300 μL/min, and 2 μL aliquot of each sample injected onto the column. TOF/MS was performed on both positive and negative ion modes. We applied the information-dependent acquisition (IDA), an artificial intelligence-based production scan mode, for detection and identification of MS/MS spectra.

#### Metabolomics data analysis

The datasets were normalized and integrated using support vector regression, then uploaded into the Metabo Analyst software for further analysis (www. metaboanalyst.ca). Datasets from both positive and negative models were log-transformed and pareto-scaled. Next, they were subjected to principal component analysis (PCA) and partial least square discriminant analysis (PLS-DA). We calculated variable importance in the projection (VIP) value, for each variable in the PLS-DA model, to determine its contribution to the classification. Metabolites with the VIP value > 1 were further analyzed using the Student t-test at the univariate level to determine the significance of each metabolite. Differences at P-value < 0.05 were considered statistically significant. The secondary metabolites screened by metabolomics were analyzed using Spearman correlation. R language and Cytoscape software were jointly used to analyze the matrix heat map, hierarchical clustering, association network, and other variables.

### Validation of expressions or regulatory roles of prioritized molecules upon mild-to-moderate COPD

The total RNA was extracted from PBMCs obtained from patients with COPD and healthy individuals using Trizol reagent (Invitrogen). It was reversely transcribed to cDNA using PrimeScript™ RT reagent Kit (TaKaRa, China). The qRT-PCR assay was performed on CFX96-C1000 system (Bio-Rad, CA) using SsoFast™ EvaGreen® supermix kit (Bio-Rad). Primers used for qRT-PCR were as follows: human CDH5: 5′-ATGAGATCGTGGTGGAAGCG-3′ (forward), 5′-TGTGTACTTGGTCTGGGTGA AG′ (reverse); human GAPDH: 5′-ACAACTTTG GTATCGTGGAAGG-3′ (forward), 5′-GCCATCACGCCACAGTTT C-3′ (reverse). The relative expression of each gene was normalized to GAPDH expression and calculated using the 2^−△△Ct^ method. Validation of metabolites (theophylline and hypoxanthine) was performed on an Ultimate 3000 UHPLC system coupled with Q-Exactive MS (Thermo Scientific).

### Statistical analysis

Data of quantitative and categorical traits were analyzed using the Mann–Whitney U and *t* tests, with *P* < 0.05 considered statistically significant. The combined values for diagnosing disease severity were calculated by binary logistic regression using a stepwise method (with a variable entered and removed if *P* < 0.05 and *P* > 0.1, respectively). The accuracy of each independent or combined indexes was determined using the auROC. The optimal threshold value was obtained by calculating the correct classification ratio (CCR). PCA seeks a linear combination of variables such that the principal components (PC) can be extracted. Loadings from the first and second PCs were used to form the weighted component scores (Y1, Y2) as a linear combination of the original 12 variables for each participant. Eigen equations showed by Y1, Y2 were combined to obtain a composite disease expression score (Y_3_ = |Y_1_ λ_1_| + |Y_2_ λ_2_|) where λ_i_ is the variance explained by each PC (eigenvalue) that accounts for most of the variation. All statistical analyses were performed using packages implemented in R (v3.2.0).

## Results

### Clinical characteristics of participants

Blood samples were collected from 70 patients with advanced COPD and 70 healthy controls from GIRD COPD Biobank (Table 1). There were statistically significant differences between COPD patients and healthy controls in terms of room ventilation, heart diseases, and pulmonary function (*P* < 0.05), but sex, smoking, pack years, age, height, weight, BMI, fan in kitchen, preserved food consumption, cooking, other comorbidities, and family history were not significantly different between the two groups (*P* > 0.05). Additional proteomics analysis was conducted using an independent validation set comprising COPD patents (n = 29) and controls (n = 31) (Table 1 on the validation stage). Results indicated that COPD patients had significantly lower mean height, weight, and pulmonary function than healthy individuals (P_max_ = 0.001). Moreover, COPD patients, but not healthy controls, reported complications related to chronic respiratory disease (CRD) at admission (*P* = 0.02). Pulmonary function (except post-FVC_%Pred) and mean platelet volume (MPV) were significantly lower in COPD patients than in healthy individuals (P_max_ = 0.025). Similarly, COPD patients exhibited significantly higher monocytes than healthy subjects (*P* = 0.027) (Table 2). Next, we used proteomics results to stratify COPD patients into 3 subgroups (Table 3): subtype I were mainly COPD without other respiratory diseases (simplex COPD, n = 19), subtype II largely for COPD co-existing with bronchiectasis (COPD-BE, n = 9), and subtype III focused on COPD co-existing with metabolic syndrome (COPD-MD, n = 12). Further analysis revealed that participants in the COPD-BE and COPD-MD groups had significantly lower room ventilation and COPD without chronic respiratory disease than the simplex COPD (*P* = 0.002 and 0.014, respectively), while those in the COPD-BE group had significantly lower pre_FEV1_%Pred relative to those in other groups (*P* = 0.025).

**Table 2:** Blood count of validation cohorts for targeted proteomics

	COPD^b^	Controls	*P*^a^
N	29	31	
Male, %	100	100	1.0
Smoking, %	100	100	1.0
Pack_years ≥ 30, %	100	100	1.0
Severe COPD, %	100	0	–
White blood cells, WBC	7.55 (1.91)	6.76 (1.8)	0.11
Neutrophil ratio	62.15 (8.25)	59.67 (8.3)	0.25
Lymphocyte ratio	24.95 (7.54)	28.98 (7.52)	0.04
Monocyte ratio	8.77 (2.27)	7.77 (2.09)	0.09
Eosinophil ratio	3.47 (2.89)	2.88 (1.66)	0.34
Basophilic cell ratio	0.66 (0.24)	0.71 (0.23)	0.44
nucleated red cells ratio	0.21 (0.72)	0.10 (0.07)	0.42
Neutrophil count	4.59 (1.47)	4.05 (1.32)	0.15
Lymphocyte count	1.85 (0.8)	1.94 (0.78)	0.66
Monocyte count^b^	0.65 (0.28)	0.52 (0.15)	**0.027**
Eosinophil count	0.26 (0.27)	0.19 (0.13)	0.24
Basophil count	0.22 (0.94)	0.04 (0.05)	0.28
NRBC	5.59 (30.08)	0.01 (0.01)	0.31
Red blood cells, RBC	4.84 (1.0)	4.80 (0.51)	0.85
Hemoglobin, HGB	141.53 (16.42)	145.3 (11.13)	0.31
Hematocrit, HCT	3.29 (11.02)	3.19 (10.43)	0.97
Mean corpuscular volume, MCV	97.17 (47.81)	94.64 (8.52)	0.77
Mean hemoglobin content, MCH	29.45 (4.33)	30.54 (3.16)	0.27
Mean hemoglobin concentration, MCHC	326.9 (10.73)	322.4 (11.9)	0.13
RDW-SD	43.94 (4.89)	43.99 (3.39)	0.96
RDW-CV	14.14 (1.08)	13.56 (1.06)	0.04
Platelet, PLT	255.9 (57.97)	236.1 (60.46)	0.20
Mean platelet volume, MPV	8.14 (1.04)	8.76 (0.94)	**0.019**
PDW	16.50 (1.2)	16.19 (1.65)	0.41
PCT	0.21 (0.04)	0.20 (0.05)	0.97

*CRD* chronic respiratory disease, *RDW-SD* standard deviation of RBC distribution width, *RDW-CV* coefficient variation of RBC distribution width, *PDW* platelet distribution width, *PCT* thrombocytocrit, *NRBC* nucleated red cells absolute value, *severe COPD* Global Initiative for Obstructive Lung Disease stage 3–4

In bold is *P* < 0.05

^a^*P* values for a two-sided χ^2^ test or t-test. Data are median (P_25_–P_75_), n (%)

^b^COPD patients exhibited significantly higher monocytes than healthy subjects

**Table 3:** Characteristics of proteomics-driven subtype cohorts by using the discovery proteomics data

	COPD			*P*^a^
	COPD	COPD-BE	COPD-MD	
N	19	9	12	
Male, %	100	100	100	1.0
Smoking, %	100	100	100	1.0
Packyears ≥ 30, %	100	100	100	1.0
Age (years), mean (SD)	59.8	67.8	63.6	0.22
Height (cm), mean (SD)	167	164	165	0.54
Weight (kg), mean (SD)	87.3	53.1	85.7	0.17
BMI (kg/m^2^), mean (SD)	32.2	18.8	32.0	0.22
Fan in kitchen, %	17 (94.4)	8 (100)	12 (100)	1.00
Good room ventilation, %	13 (72.2)	1 (12.5)	2 (16.7)	**0.002**
Often preserved food, %	3 (16.7)	0	0	0.54
Often cook, %	5 (27.8)	3 (37.5)	3 (25)	0.88
Comorbidity, %				
CRD	6 (31.6)	3 (33.3)	4 (33.3)	0.99
Hypertension	8 (42.1)	3 (33.3)	5 (41.7)	0.90
Diabetes	4 (21.1)	1 (11.1)	1 (8.3)	0.85
Heart diseases	6 (31.6)	3 (33.3)	5 (41.7)	0.84
Stroke	2 (10.5)	0	0	0.71
Family history, %				
Cancer	3 (15.8)	0	0	0.30
CRD without COPD	6 (42.8)	1.0 (11.1)	0	**0.014**
Severe COPD%	100	100	100	1.0
pre_FVC_%Pred	81.1 (29.5)	53.2 (12.0)	63.4 (19.7)	0.19
pre_FEV1_%Pred	69.8 (31.5)	24.1 (4.4)	41.0 (16.9)	**0.025**
pre_FEV1/FVC_%Pred	81.8 (29.5)	53.2 (12.1)	63.4 (19.7)	0.19
post_FVC_%Pred	77.5 (28.4)	64.0 (4.4)	71.7 (17.6)	0.69
post_FEV1_%Pred	66.1 (29.1)	29.3 (7.3)	44.1 (18.1)	0.07
post_FEV1/FVC_%Pred	77.5 (28.4)	64.0 (4.4)	71.7 (17.6)	0.69

Bold values indicate significant differences

*CRD* chronic respiratory disease, *severe COPD* Global Initiative for Obstructive Lung Disease stage 3–4, *COPD-BE* COPD co-existing with bronchiectasis, *COPD-MD* COPD co-existing with metabolic syndrome

^a^*P-*values for a two-sided χ^2^ test or oneway-ANOVA, or Fisher’s exact test, non-parametric test.as appropriate. Data are median (P_25_–P_75_), n (%)

### Proteomic profiles and functional alterations related to COPD

Serum samples were obtained from 40 patients with advanced COPD and 40 healthy controls for TMT-labeled proteomic analysis. The proteomic patterns of serum from COPD patients were distinct from those of serum obtained from healthy controls. A total of 1432 proteins were identified and quantified. Quality control analysis was carried out, the lengths and mass errors of peptides, as well as coverage and sequence distribution of the proteins were calculated (Additional file 1: Fig. S1A–D). Consequently, 251 differentially expressed proteins (DEPs) were identified between the two groups, of which 151 and 100 were significantly up-regulated and down-regulated, respectively (fold change ≥ 1.2 or ≤ 0.83 and a *P* < 0.05) (Additional file 1: Fig. S1E). Moreover, 31.43% of these proteins were involved in extracellular matrix, whereas 29.29 and 18.57% among them regulated functions in the cytoplasm and nucleus, respectively (Additional file 1: Fig. S1F). The DEPs were divided into Q1–Q4 according to the multiple of fold change, and the heatmap of enrichment analysis (GO and KEGG) shown in Additional file 2: Fig. S2. In sum, the quality control analysis showed the data were acquired with a high degree of consistency and reproducibility, and the significantly up-regulated DEPs indicated a generally activated effect of biological processes in COPD.

**Fig. 2:**
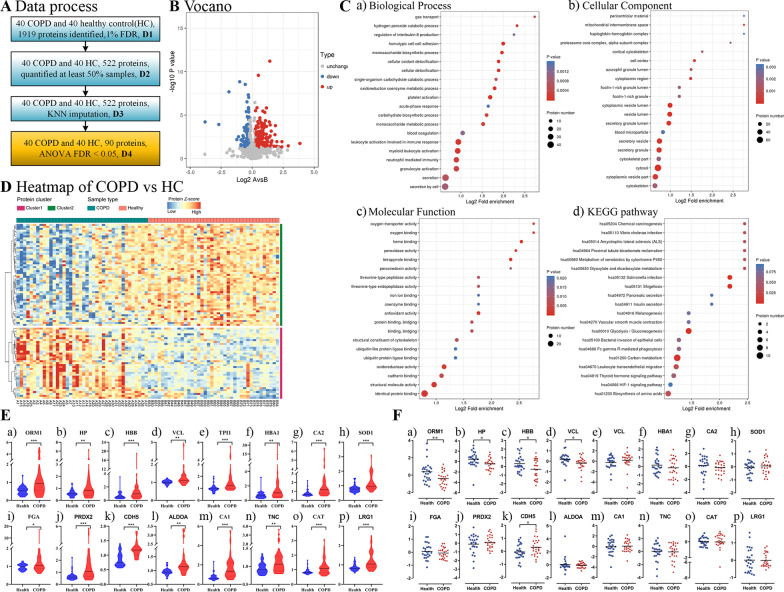
Proteomic profiles and functional alterations related to COPD. **A** Data process. **B** Venn plot showing identification of the COPD specific proteins among COPD vs healthy controls. **C** Gene Ontology annotation and KEGG enrichment analysis of differentiated expressed proteins (DEPs). **D** Heatmap showing the differentiated expressed proteins (DEPs). The red and colors in the heatmap denote higher gene expression and lower gene expression, respectively. **E** The final selected dysregulated proteins. **F** Protein validation via targeted proteomics (PRM)

The detailed data processing protocols for COPD and healthy controls are showed in Fig. 2A. In total, 251 dysregulated DEPs were identified between the two groups (Fig. 2B). For biological processes, these proteins were mainly involved in immune response, myeloid leukocyte activation, neutrophil mediated immunity, granulocyte activation, platelet activation, and homotypic cell–cell adhesion. For molecular functions, the proteins were primarily involved in seven function processes, namely identical protein binding, structural molecule activity, cadherin binding, oxidoreductase activity, structural constituent of cytoskeleton, tetrapyrrole binding, and heme binding. Most of these proteins were located in vesicle lumen and secretory granule (Fig. 2C). Results from KEGG pathway analyses revealed that these DEPs were significantly enriched in carbon metabolism, and glycolysis/gluconeogenesis. Heatmap analyses showed higher antioxidant activity and activated glycosaminoglycan binding in COPD compared to healthy controls (Fig. 2D). Among these DEPs, the final dysregulated proteins were selected (Fig. 2E), and validated by targeted proteomics according to differential significance levels, including ORM1, HP, HBB, VCL, TPIA, HBA1, CA2, SOD1, FGA, PRDX2, CDH5, ALDOA, CA1, TNC, CAT, and LRG1 (Fig. 2F). In final, 16 DEPs were selected associated with COPD compared with healthy controls.

### Protein validation via targeted proteomics (PRM)

The COPD-related proteomics and functional alteration results from the discovery study were then used to develop protein marker panels for accurate prediction of severity of COPD. Thus, we analyzed members of upregulated functional groups based on top *P*_min_ value and fold changes (max). Finally, 16 DEPs with confident quantitation data were validated in an additional cohort comprising 29 COPD and 31 healthy controls (Additional file 6: Table S1). Considering the challenge of quantifying dozens of protein candidates in parallel, we employed a median-throughput mass spectrometry-based approach as the Parallel Reaction Monitoring (PRM) for analysis of 176 tryptic peptides. Eventually, this targeted proteomic analysis approach detected 16 protein candidates with robust signal across the validation set. The trends of the marked proteins in COPD samples corroborated results from the discovery study (Fig. 2F). To sum to, the final 5 significantly dysregulated proteins were selected after validating via targeted proteomics, including ORM1, HP, HBB, VCL, and CDH5.

### Proteomic subtypes of COPD and their association with clinical outcomes

**Fig. 3:**
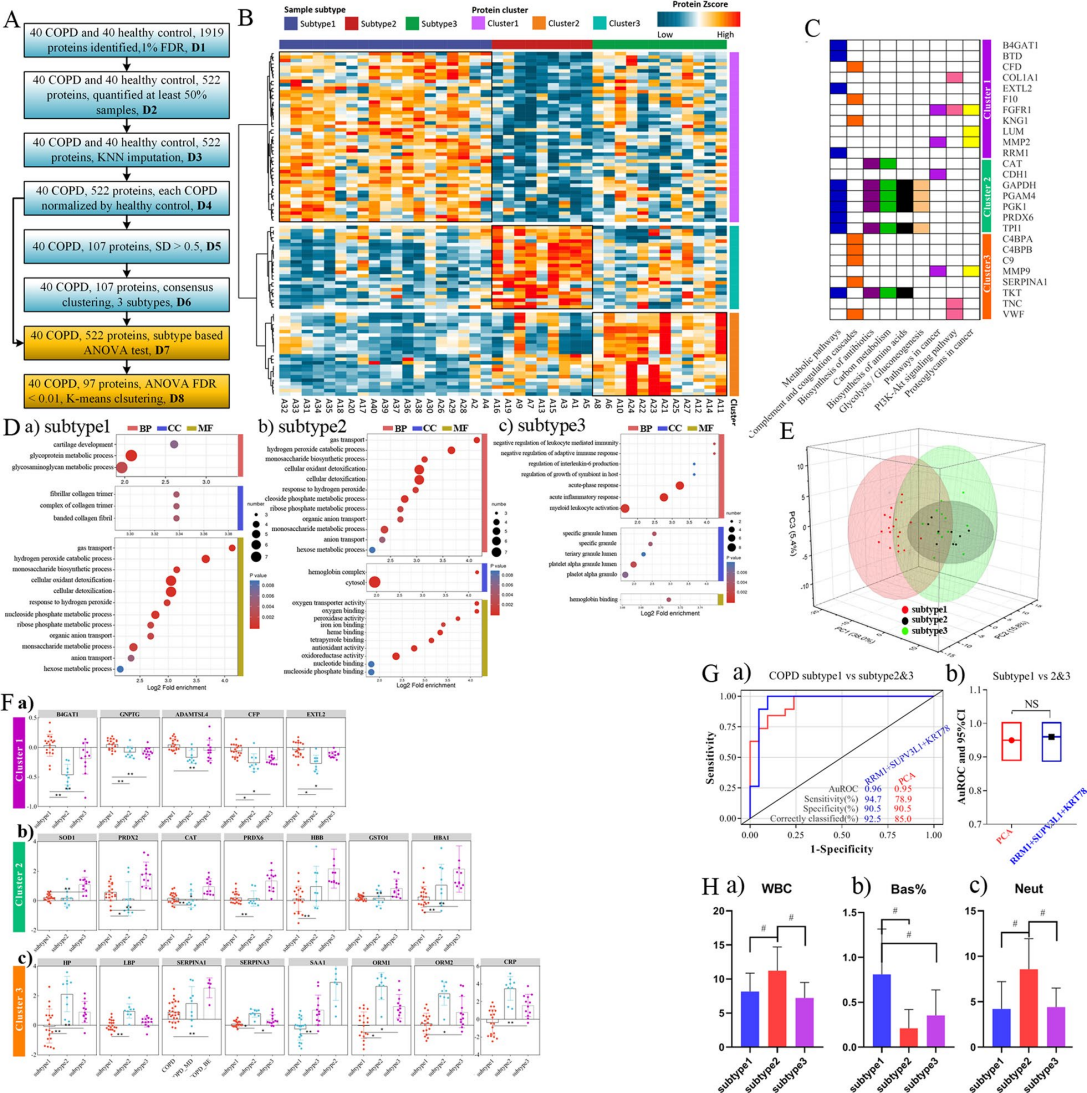
Proteomic subtypes of COPD and their association with clinical outcomes. **A** Data process. **B** Heatmap showing the DEPs among COPD 3 subtypes. Proteome based stratification of COPD revealed three subtypes (subtype I–III) related to different clinical outcomes and molecular feature: subtype I were patients with simplex COPD, and subtype II were COPD mainly co-existing with bronchiectasis, and subtype III were COPD largely co-existing with metabolic syndrome. The red and colors in the heatmap denote higher gene expression and lower gene expression, respectively. **C** Pathways for dysregulated proteins enriched. **D** Gene Ontology annotation and KEGG enrichment analysis of DEPs among COPD 3 subtypes. **E** A tridimensional plot via Principal Component Analysis (PCA) showing the configuration of indexes on COPD and its co-morbidities. **F** The final selected dysregulated proteins. **G** ROC analysis of PCA and the combination of (RRM1, SUPV3L1, KRT78). **H** The corresponding information on blood tests

Consensus clustering based on the 107 most variable proteins in COPD identified three proteomic subtypes (each disease normalized by health and SD > 0.5) (Fig. 3A). They were designated as subtype I (n = 19), subtype II (COPD-BE, n = 9), and subtype III (COPD-MD, n = 12). The resulting heatmap revealed that the DEPs were significantly enriched in metabolic pathways and complement and coagulation cascades in subtype I. Moreover, 5 highly expressed proteins, including B4GAT1, GNPTG, ADAMTSL4, CFP, and EXTL2 were identified. We found that the subtype II was enriched in metabolic pathways, biosynthesis of antibiotics, carbon metabolism, biosynthesis of amino acids, and glycolysis/gluconeogenesis, and the involved proteins included SOD1, PRDX2, CAT, PRDX6, HBB, GSTO1, and HBA1. For subtype III group, the complement and coagulation cascades were significantly enriched, and the following proteins were enriched: HP, LBP, SERPINA (1, 3), SAA1, CRP, ORM1, ORM2, and CRP. GO enrichment analysis was performed to annotate the putative functional implications of the grouped DEPs (Fig. 3B–D, F).

A tridimensional plot via PCA showed the configuration of indexes on COPD and COPD with co-morbidities in Fig. 3E. Plots of individual component scores for the first principal component (PC1) versus the second principal component (PC2) versus the third principal component (PC3) were provided. PC1, PC2, and PC3 showed clear separation of COPD from COPD subtypes. Combinations of PC1, PC2, and PC3 could explain 58.4% proportion of the whole variances. Based on the selected proteins panel, as indicated in Fig. 3G, ROC analysis of PCA and the combination of RRM1 + SUPV3L1 + KRT78 was calculated, and results showed that the auROC was 0.95 and 0.96, respectively. There was no significant difference between PCA analysis and the combination of RRM1 + SUPV3L1 + KRT78 (P > 0.05). In addition, basophil count showed the ability to distinguish COPD from COPD-BE or COPD-MD, while white blood cells and neutrophil ratio was able to distinguish COPD from COPD-BE, as well COPD-BE from COPD-MD (Fig. 3H).

In sum, COPD were subtyped into three based on their corresponding clinical outcomes. We also identified that both PCA analysis and the combination of RRM1 + SUPV3L1 + KRT78 could effectively differentiate COPD and COPD with co-morbidities.

### Metabolomic profiles and functional alterations associated with COPD

**Fig. 4:**
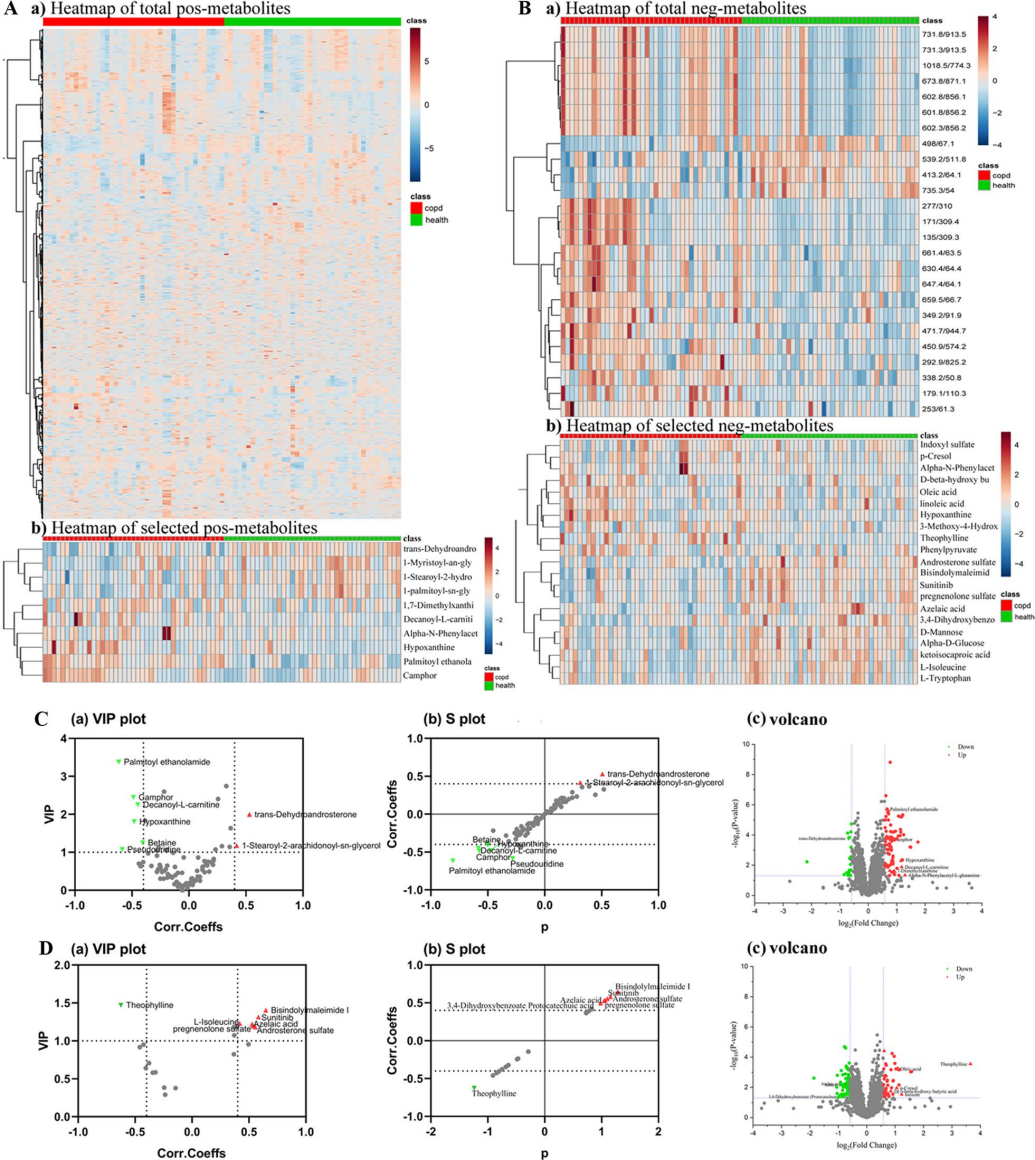
Metabolomic profiles and functional alterations associated with COPD. **A** Heatmaps depicting clustering of total and selected metabolites across positive modes. **B** Heatmaps depicting clustering of total and selected metabolites across negative modes. **C** The functions of the selected positive metabolites depicted using variable importance in the projection (VIP) value and volcano. **D** The functions of the selected negative metabolites depicted using VIP and volcano

A library of known metabolite standards (APPLIED PROTEINS TECHNOLOGY Co. Ltd) was employed to identify 210 differentially expressed metabolites (DEMs) in COPD compared to healthy controls. In addition, quality control analyses were carried out based on correlation distributions for total and separately metabolites (or by group). The EBAM plots, normalization, PLS-DA, and t test were conducted (Additional file 3: Fig. S3A, B). Results indicated that PLS-DA produced a model that could separate positive and negative metabolites. Heatmaps depicting clustering of total and selected metabolites in positive and negative modes, respectively, are shown in Fig. 4A, B. Notably, 44 differentially expressed metabolites between the two groups were identified, among which 15 and 29 were positive and negative metabolites, respectively. The functions of the selected metabolites were displayed on VIP and volcano plots, and these metabolites were palmitoylethanolamide, trans-Dehydroandrosterone, decanoyl-l-carnitine, betaine, pseudouridine, camphor, 1-stearoyl-2-hydroxy-sn-glycero, hypoxanthine, theophylline, l-isoleucine pregnenolone, androsterone sulfate, azelaic acid, sunitinib, bisindolymalemide1 (Fig. 4C, D). By using the complementary approach, the weighted gene co-expression network analysis (WGCNA), we identified several co-expression modules (Additional file 5: Fig. S5). The Betaine in MEcyan module was found to be significantly associated with COPD. Summary, 8 positive- and 6 negative-metabolites were selected by metabolomic analysis.

### Integrated analyses of proteomics and metabolomics data

#### Correlation analysis

**Fig. 5:**
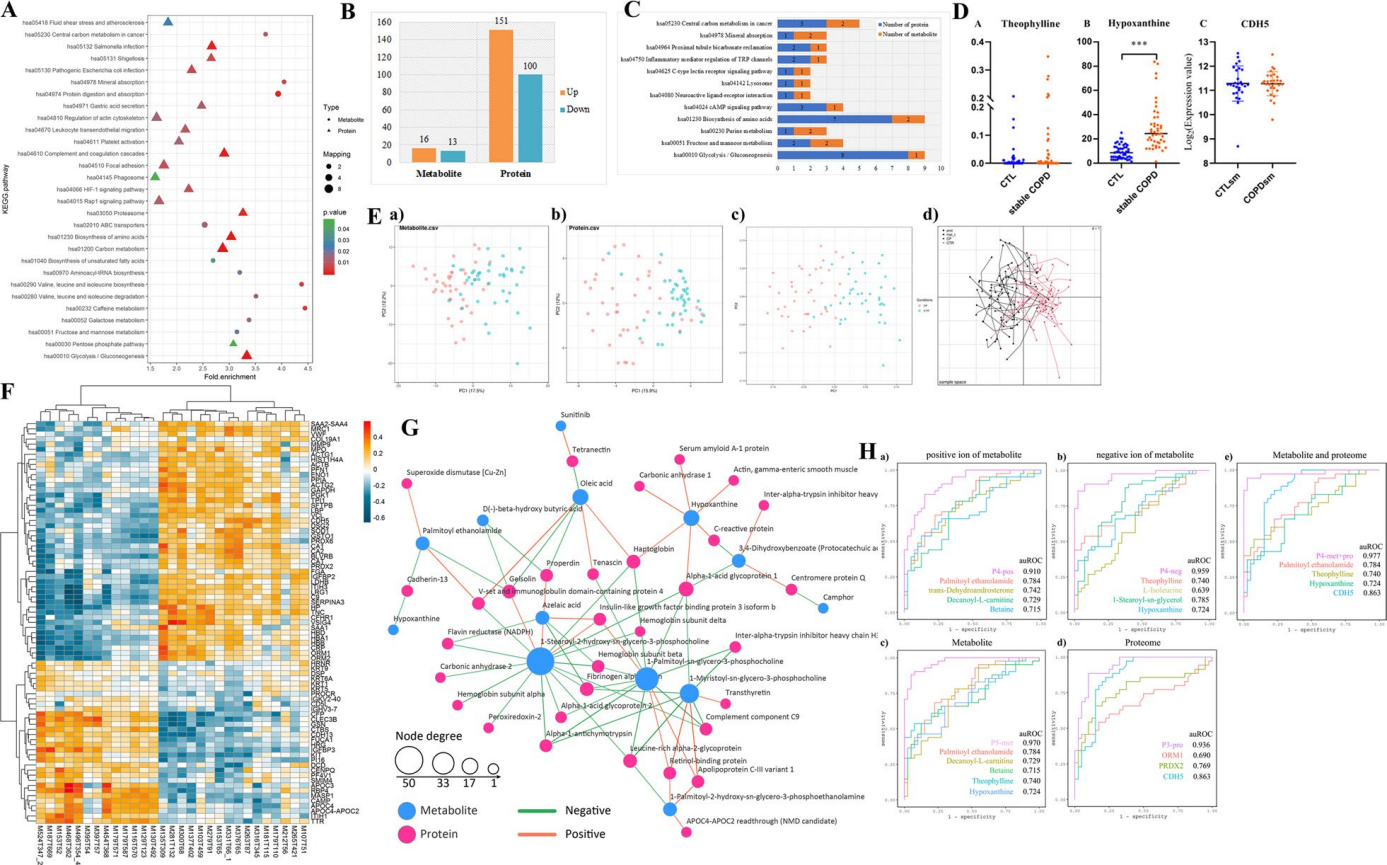
Integrated analyses of proteomics and metabolomics data. **A** KEGG enrichment analysis of differentiated expressed proteins (DEPs) and differentiated expressed metabolites (DEMs). **B** The number of DEPs and DEMs. **C** Number of proteins and metabolites common involved in one pathway. **D** Validation study of the final predicted model on mild-to-moderate COPD patients. **E** PCA analysis of proteomics or (and) metabolomics data. **F** Heatmap analyses of DEPs and DEMs identified relatively strong or weak proteins-metabolites correlations. **G** The network analysis between DEPs and DEMs. **H** Establishment of predictive panels for COPD (single and combined biomarkers analysis)

After appropriate sample quality control (QC) and normalization procedures, we performed PCA on the proteomics and metabolomics data. All datasets effectively distinguished COPD from healthy controls, with the best separation observed with the combined proteomics and metabolomics analysis (Fig. 5E). We found that a considerable number of proteins and metabolites were both involved in mineral absorption, proximal tubule bicarbonate reclamation, inflammatory mediator regulation, lysosome, neuroactive ligand-receptor interaction, cAMP signaling pathway, biosynthesis of amino acids, purine metabolism, fructose and mannose metabolism, glycolysis/gluconeogenesis Fig. 5C. However, *P* value of 0.05 as a cutoff, the significant enrichment pathways were enriched including both proteomics and metabolomics data in Fig. 5A, B. Heatmap analyses of the differentially expressed proteins and metabolites identified relatively strong or weak proteins-metabolites correlations. Proteins or metabolites with strong or weak correlations were detailed in Fig. 5F. The final differential proteins (DEPs) or DEMs were selected as the target proteins or metabolites. To analyze the interactions between them, the network between DEPs and DEMs was analyzed by Cytoscape, and the results detailed in Fig. 5G. In sum, enrichment analyses of DEPs and DEMs were performed to investigate the potential correlations between them, and the results showed that there were strong or weak correlations between these proteins or metabolites.

#### Establishment of diagnostic panels for COPD (diagnostic efficacy of single biomarkers)

Before the biomarkers were integrated, the profile of each biomarker was first analyzed separately (Fig. 5Ha, Hb). Subsequently, the ROC models were applied to calculate the auROC, specificity, and sensitivity of single biomarkers. The calculations were performed using the following formula: %sensitivity = [true-positive/(true-positive + false-negative)] * 100; %specificity = [true-negative/(true-negative + false-positive)] * 100. Thereafter, 7 positive and 7 negative metabolites, alongside 6 proteins that had shown significant changes in COPD patients (P_max_ = 0.029) were individually subjected to ROC analysis, to evaluate their sensitivity and specificity and help discriminate COPD from healthy controls. As showed in Additional file 7: Table S2, results indicated that palmitoylethanolamide, which was used as a positive metabolite had a maximal auROC of 78.0%, with sensitivity and specificity of 68.0 and 72%, respectively. On the other hand, 1-Stearoyl-sn-glycerol (used as a negative metabolite) had an auROC of 78.0% and a sensitivity of 71.0% against controls. For the proteomics data, CDH5 recorded a maximum auROC of 85.0%, with a sensitivity and specificity of 80.0 and 78%, respectively. Results from blood routine tests showed that MPV had an auROC of 64.0%, with a sensitivity and specificity of 59.0 and 53.0% respectively, while monocytes recorded an auROC of 68.0%, with a sensitivity of 62.0%, and specificity of 63.0%. In sum, diagnostic efficacy of single biomarker was established based on metabolites, proteins, and blood routine test. The result indicated that palmitoylethanolamide, 1-Stearoyl-sn-glycerol, and CDH5 had the highest auROC values for positive metabolites, negative metabolites, and proteins, respectively.

#### Diagnostic capability of combined biomarkers

The data shown in Additional file 8: Table S3 and Fig. 5H indicate that analysis of predictive capability of a combination of 4 positive metabolites, namely palmitoylethanolamide, *trans*-dehydroandrosterone, decanoyl-l-carnitine, and betaine, obtained an auROC of 91.0%, with a sensitivity and specificity of 83.0 and 85.0%, respectively. When 4 negative metabolites (theophylline, l-isoleucine, 1-stearoyl-sn-glycerol, and hypoxanthine) were combined, an auROC of 95.9%, was obtained with a sensitivity of 90.0%, and specificity of 90.0%. On the other hand, combining scores from 3 positive metabolites (palmitoylethanolamide, decanoyl-l-carnitine, and betaine) with those from 2 negative ones (theophylline and hypoxanthine) resulted in ROC curve with an auROC of 97.0%, a sensitivity of 88.0%, and specificity of 93.0%. The same model was used to construct a logistic model using the 5 markers, dubbed diagnostic P5, and observed differential abundance in predicting serious COPD as follows:

$${\text{Y}}_{{({\text{COPD}} = 1|{\text{control}} = 0)}} = - 14.645 + \left( {0.41*{\text{palmitoylethanolamide}} + 1.41*{\text{decanoyl-L}}\;{\text{carnitine}} - 4.83*{\text{betaine}} + 0.15*{\text{theophylline}} + 1.17*{\text{hypoxanthine}}} \right)/10000.$$

Using this P5 score, advanced COPD participant can be distinguished predicted with high sensitivity and specificity, and the auROC reached 0.97 in our data set (Fig. 5Hc).

Combining scores from all proteins resulted in an auROC of 93.6%, with a sensitivity and specificity of 88.0 and 90.0%, respectively (Fig. 5Hd). The 3-protein (ORM1, CDH5, and PRDX2) based logistic model generated a dichotomous score, dubbed diagnostic P3, which allowed classification of each participant. The relationship between the probability score of a participant being positively diagnosed with advanced COPD and the log_2_ intensity value of each protein marker was defined as follows:

$${\text{Y}}_{{({\text{COPD}} = 1|{\text{control}} = 0)}} = - 10.323 + 2.354*{\text{ORM}}1 + 6.834*{\text{CDH}}5 + 1.694*{\text{PRDX}}2.$$

Combining scores from 3 metabolites and that of 1 protein resulted in a high auROC value of 98.0%, with a sensitivity of 94.0%, and specificity of 95.0% (Additional file 9: Table S4). The final logistic model, dubbed P4, comprised palmitoylethanolamide, theophylline, hypoxanthine, and CDH5 (all auROC_min_ > 0.724), and was expressed as follows:

$${\text{Y}}_{{({\text{COPD}} = 1|{\text{control}} = 0)}} = - 17.934 + \left( {0.46*{\text{palmitoylethanolamide}} + 0.13*{\text{theophylline}} + 0.77*{\text{hypoxanthine}}} \right)/10000 + 8.340*{\text{CDH}}5.$$

The scores from the P4 model had significantly higher power than scores from other models in predicting advanced COPD. The sensitivity, specificity, and auROC of P4 for COPD prediction were greatest (Fig. 5He). The highest Youden index (0.835), which indicates the model’s ability to correctly diagnose true serious COPD patients, was achieved at the cut-point. Taken together, results from the logistic model indicated that a combination of palmitoylethanolamide, theophylline, hypoxanthine, and CDH5 was the best signature of serum biomarkers for predicting advanced COPD.

#### Validation study of the final predicted model

The final predictors were further verified on mild-to-moderate COPD patients and healthy controls. For CDH5, it was found that its expression was not significantly different between COPD and controls (Fig. 5D). The clinical and demographic characteristics of participants are presented in Table 4. A total of 30 patients with COPD and 30 healthy controls were enrolled. Of note, there were statistically significant differences between COPD patients and controls in terms of pack_years, age, heart disease, and pulmonary function (*P* < 0.05), but no significant difference was found between the two groups in terms of sex, smoking, BMI, respiratory symptoms, chronic respiratory diseases, poison exposure, room ventilation, cook, other comorbidities, and family history of cancers (*P* > 0.05). Among the metabolites, theophylline was not significantly different between the two groups, but hypoxanthine showed significant differences in the validation cohort (data missing for palmitoylethanolamide) (Fig. 5D). The detailed clinical and demographic characteristics for participants was described in the previous study [36].

**Table 4:** Characteristics of validation cohorts on mild-to-moderate COPD for CDH5

	Controls	COPD^b^	*P*^a^
N	30	30	–
Male, %	96.67	83.33	0.20
Smoking, %	93.33	76.67	0.15
Pack_years, median (IQR)	25 (14, 40)	40 (20, 50)	**0.01**
Age (years), mean (SD)	59.97 ± 4.66	65.0 ± 7.00	**< 0.01**
BMI (kg/m^2^), mean (SD)	23.97 ± 3.86	22.36 ± 2.59	0.06
Cough without having a cold	7 (23.33)	14 (46.67)	0.06
Phlegm without having a cold	10 (33.33)	13 (43.33)	0.43
Chronic respiratory diseases	3 (10.00)	9 (30.00)	0.05
Poison exposure	14 (46.67)	14 (46.67)	1.00
Good room ventilation, %	19 (63.33)	19 (63.33)	1.00
Offen cook, %	10 (33.33)	15 (50.00)	1.00
Comorbidity, %			
Hypertension	9 (30.00)	8 (26.67)	0.77
Diabetes	2 (6.67)	2 (6.67)	1.00
Heart disease	0	5 (16.67)	**0.02**
Stroke	2 (6.67)	1 (3.33)	0.55
Family history of cancer, %	8 (26.67)	11 (36.67)	0.41
Pulmonary function, mean (SD)			
FEV1_%Pred	100.51 ± 13.12	68.66 ± 6.90	**< 0.01**
FEV1/FVC_%Pred	80.25 ± 3.68	62.23 ± 6.53	**< 0.01**

Bold values indicate significant differences

*CRD* chronic respiratory disease

^a^*P-*values for a two-sided χ^2^ test or t-test. Data are median (P_25_–P_75_), n (%)

^b^Mild-to-moderate COPD, Global Initiative for Obstructive Lung Disease stage 1–2

In sum, theophylline and CDH5 had not significantly different between mild-to-moderate COPD patients and healthy controls.

## Discussion

Globally, COPD kills more than 3 million people every year. Although several advances have been achieved in the symptomatic treatment and prevention of acute clinical cases, there are few interventions for ameliorating disease progression or decrease mortality. Therefore, it is important to identify biomarkers that can predict disease occurrence or aid in diagnose of advanced COPD. This will facilitate early intervention and prevent progression. In this study, we found that a combination of theophylline, palmitoylethanolamide, hypoxanthine, and CDH5 provides a high diagnostic accuracy. Proteomics facilitates the differentiation of COPD from COPD with co-morbidities. We also found that basophil count could effectively distinguish COPD from COPD-BE or COPD-MD. Moreover, hypoxanthine was still significantly different between mild-to-moderate COPD and controls.

In clinical practice, plasma or serum is the most widely used specimen for biomarker discovery because proteins/metabolites in the circulatory system likely reflect disease pathophysiology. Our dataset can be used to identify potential predictive biomarkers of advanced COPD. Theophylline and the other three methylxanthine derivatives (aminophylline, etophylline, and caffeine), are the first four compounds to have been approved for use in clinical practice [12]. Among them, as bronchodilators, theophylline is the most effective and is widely used for the treatment of asthma and COPD. Evidences showed that corticosteroids and theophylline, both in low doses, have synergistic and clinically useful anti-inflammatory effects in COPD [26]. The underlying molecular mechanisms suggest that this happens through theophylline increasing the activity of the nuclear enzyme histone deacetylase-2 (HDAC2), which is decreased in COPD, therefore preventing the anti-inflammatory effect of corticosteroids [1]. Scientists have identified that low-dose theophylline, especially below those which lead to bronchodilatation, can reverse corticosteroid insensitivity in COPD [9, 26]. Another study has demonstrated an effect for low-dose theophylline on the forced expiratory volume in one second (FEV1) as well as exacerbations [37]. The metabolic disposition of theophylline in humans was first reported by Brodie et al. [3]. Following a therapeutic dose, only 85% has been accounted for by measurement of known metabolites, and unchanged drug excreted in urine. Therefore, about 10% of theophylline administered to man appears in urine in an unchanged form. This would be one of the main sources of theophylline in the body, and the main reason for deviations between patients and controls. It may also explain why there were no differences in theophylline between mild-to-moderate COPD and control in the present study. In addition, as one of the methylxanthines, theophylline is also a natural and synthetic compound found in tea, most of which is metabolized by some types of bacteria and fungi, some of which exist in blood circulation in the human body [35]. However, the information about tea drinking was lacking in this study. This need to be investigated in the future.

Hypoxanthine is a product of ATP degradation, and its conversion to uric acid is facilitated by the enzyme xanthine oxidase, generating free oxygen radicals [5]. It is a metabolite that is involved in purine biosynthesis and nucleotide metabolism, and often serves as a biomarker. For instance, hypoxanthine is a potential marker for oxidative stress in cystic fibrosis [31]; a combination of eight metabolites including uric acid, stearic acid, threitol, acetylgalactosamine, heptadecanoic acid, aspartic acid, xanthosine and hypoxanthine were found to accurately diagnose asthma while discriminating between healthy control and asthma subgroups. In preschool children with cystic fibrosis, hypoxanthine concentrations were found to be elevated in BALF from lobes of the lung containing localized bronchiectasis and were correlated with neutrophil counts and important clinical outcomes [7, 32]. Elevated hypoxanthine concentrations in various body fluids are as a result of vital tissue hypoxia. For mild-to-moderate COPD, higher level of hypoxanthine has also been demonstrated, and this might explain that tissue hypoxia exists in COPD at early time. Suppressed serum hypoxanthine levels have been reported in lung cancer [14] and cystic fibrosis lung disease [17]. Increased conversion to uric acid during exacerbation, may result in a reduction in the concentration of hypoxanthine, generating superoxide and hydroxyl radicals in which cause cellular damage. However, this phenomenon needs to be investigated in COPD.

Vascular endothelial cadherin 5 (CDH5), an endothelial specific cell–cell adhesion molecule, plays important roles in the formation, maturation, and remodeling of the vascular wall [10]. RAB26 is a newly identified small GTPase involved in regulation of endothelial cell (EC) permeability [6]. It confers protective effects on EC permeability, which is in part dependent on autophagic targeting of active SRC, and the resultant CDH5 dephosphorylation maintains adherent junction stabilization. During inflammation, CDH5 phosphorylation at tyrosine residues induces opening of endothelial adherent junctions [30]. Post-translational modifications of CDH5 at tyrosine residues are involved in vascular permeability and leukocyte transmigration. Moreover, cell surface CDH5 phosphorylation is directly linked to EC barrier integrity. These results suggest that any change in CDH5 will impact endothelial barrier functions at multiple levels and CDH5 inhibition may lead to a marked increase in permeability [11, 27]. Enhanced permeability is an early step in the angiogenic process, enabling endothelial migration out of the primary vessel in order to format the tumor neovasculature in the next [18]. Moreover, induction of CDH5 during epithelial mesenchymal transformation accentuates breast cancer progression via TGF-β signaling, indicating that in certain tumor cells, CDH5 can induce cellular responses that counteract its inhibitory role in cell–cell contact growth in EC [16]. Therefore, CDH5 has two functions in angiogenesis and cancer progression. Smoking, a key factor that regulates COPD development, causes hypoxia, which is an important driver of angiogenesis which participates in the pathogenesis of COPD.

COPD is a heterogeneous condition that presents the opportunity for precision therapy based on more precise disease subtypes. Subtype directed therapies, such as inhaled corticosteroids for patients with frequent exacerbations, have had only moderate success. This is likely due to imprecise phenotype categorization, the limited number of drugs for treating COPD, and the generally modest effects of most of these drugs. It is, therefore, crucial to provide precise therapies for patients with specific COPD subtypes based on specific biomarkers. Since comorbidities have a tremendous impact on the prognosis and severity of COPD, the 2015 American Thoracic Society/European Respiratory Society (ATS/ERS) Research Statement on COPD urgently called for studies to elucidate on the pathological mechanisms involved in the association between COPD and its comorbidities. Since comorbidities have influence the clinical outcomes of COPD, identification of the mechanisms linking COPD to its comorbidities is key to developing effective therapies. Presently, it has not been established whether BE or MD is an independent co-existing condition or a direct consequence of progressive lung pathology in COPD patients. In this study, we developed a pipeline for proteomic dominated subtyping of COPD, which complements subtyping approaches based on clinical or imaging data [23, 29], as well clustering by omics in Chinese. In particular, based on proteomics results, COPD patients were grouped into three clusters according to prominent molecular features, including simplex COPD, COPD-BE, and COPD-MD. To further differentiate the disease subtypes, we identified that COPD-MD is highly involved in complement and coagulation cascades processes, and was enriched with various proteins, including HP, LBP, SERPINA1, SERPINA3, SAA1, ORM1, ORM2, and CRP. COPD-BE participates in complement and coagulation cascades processes, and is enriched with various proteins, including metabolic pathways, biosynthesis of antibiotics, carbon metabolism, biosynthesis of amino acids, and glycolysis/gluconeogenesis. Moreover, SOD1, PRDX2, CAT, PRDX6, HBB, GSTO1, and HBA1 were highly expressed in COPD-MD. Since advanced COPD possess unique metabolic pathways and typically express protein isoforms that may have special functions, proteomic approaches for studies of metabolic pathways are especially important.

This study has some limitations. First, no follow-up investigation of the same participants was carried out. Further multi-center and longitudinal studies are need to the prediction performance of the identified biomarkers in advanced COPD. Second, this was a retrospective study, therefore, laboratory tests might be underestimated in medical records, making it difficult to explore their effects on outcomes. Moreover, information on medication, disease control status, and disease phenotypes before admission were incomplete. The impact of these factors on disease expression should be further evaluated. Third, the study population was relatively small. Thus, large prospective studies should be performed to validate the present findings. Finally, although traditional methods, such as logistic regression used in this study, are often used to establish prediction models, it has been suggested that Artificial Intelligence (AI) based machine learning (ML) approaches may be more accurate than traditional logistic regression. This is because AI-based ML can overcome many of the disadvantages of conventional statistical approaches used for analyses of high-volume next generation sequencing data. For instance, ML does not require full details of sequencing measurements and can extract features from sequences [28]. Therefore, ML approaches should be considered in further studies.

## Conclusion

An integrated array of proteins and metabolites, including theophylline, palmitoylethanolamide, hypoxanthine, and CDH5 showed the potential to diagnose advanced COPD patients with a high accuracy. Based on proteomics, advanced COPD patients were assigned into 3 subgroups. In particular, COPD-MD was found to be highly involved in complement and coagulation cascades processes, and HP, LBP, SERPINA1, SERPINA3, SAA1, ORM1, ORM2, and CRP were highly enriched.

## Supplementary Information

**Additional file 1: Figure S1.** Quality control analysis of proteomic data. A. The lengths of peptides. B. The mass errors of peptides. C. Protein mass. D. Coverage and sequence distribution of the proteins. E. Number of proteins in comparable groups. F. Protein subcellular distribution.

**Additional file 2: Figure S2.** Heatmap showing of the DEPs analysis on GO and KEGG. The DEPs were divided into Q1–Q4 according to the multiple of fold change, and the heatmap of enrichment analysis preformed on GO and KEGG.

**Additional file 3: Figure S3.** Quality control analysis of metabolomic data. Correlation distributions for positive and negative metabolites, respectively, and EBAM plots, normalization, PLS-DA, and t test generated.

**Additional file 4: Figure S4.** Correlation distributions for total and selected-metabolites. Correlation distributions for total and selected-metabolites grouped by COPD and healthy controls.

**Additional file 5: Figure S5.** Metabolomics data analysis on the weighted gene co-expression network analysis (WGCNA) in COPD compared to healthy controls. A, Positive metabolites; B, Negative metabolites.

**Additional file 6: Table S1.** Proteins was generated for the targeted proteomic survey.

**Additional file 7: Table S2.** Predictive efficacy of single biomarker.

**Additional file 8: Table S3.** Predictive efficacy of the combined biomarkers.

**Additional file 9: Table S4.** Predictive efficacy of the combined biomarkers for the best serum biomarkers.

## Data Availability

The datasets used during the current study are available from https://www.ncmi.cn/phda/projectDataDetail.html?id=f7758371-0fe3-35e8-b428-dd27b6940451.

## Abbreviations

COPD: Chronic obstructive pulmonary disease
PCA: Principal Component Analysis
CDH5: Cadherin 5
RRM1: Ribonucleotide reductase catalytic subunit M1
SUPV3L1: Suv3 like RNA helicase
KRT78: Keratin 78
CRD: Chronic respiratory disease
COPD-BE: COPD co-existing with bronchiectasis
COPD-MD: COPD co-existing with metabolic syndrome
MPV: Mean platelet volume
DEPs: Differentially expressed proteins
GO: Gene Ontology
KEGG: Kyoto Encyclopedia of Genes and Genomes
HP: Haptoglobin
HBB: Hemoglobin subunit beta
VCL: Vinculin
HBA1: Hemoglobin subunit alpha 1
CA1/2: Carbonic anhydrase 1/2
SOD1: Superoxide dismutase 1
FGA: Fibrinogen alpha chain
PRDX2: Peroxiredoxin 2
ALDOA: Aldolase, fructose-bisphosphate A
TNC: Tenascin C
CAT: Catalase
PRM: Parallel Reaction Monitoring
B4GAT1: Beta-1,4-glucuronyltransferase 1
GNPTG: *N*-Acetylglucosamine-1-phosphate transferase subunit gamma
ADAMTSL4: ADAMTS like 4
CFP: Complement factor properdin
EXTL2: Exostosin like glycosyltransferase 2
SAA1: Serum amyloid A1
CRP: C-reactive protein
ORM1/2: Orosomucoid 1/2
DEMs: Differentially expressed metabolites
WGCNA: Weighted gene co-expression network analysis
BALF: Bronchoalveolar lavage fluid
RAB26: Member RAS oncogene family
SERPINA1: Serpin family A member 1
GOLD: Global Initiative for Obstructive Lung Disease
PBMCs: Peripheral blood mononuclear cells
