# Supplementary figures and images for: Proteomics and metabolomics profiling reveal panels of circulating diagnostic biomarkers and molecular subtypes in stable COPD
Source: Respir Res. 2023 Mar 11;24:73. doi: 10.1186/s12931-023-02349-x (PMC10007826; doi:10.1186/s12931-023-02349-x)

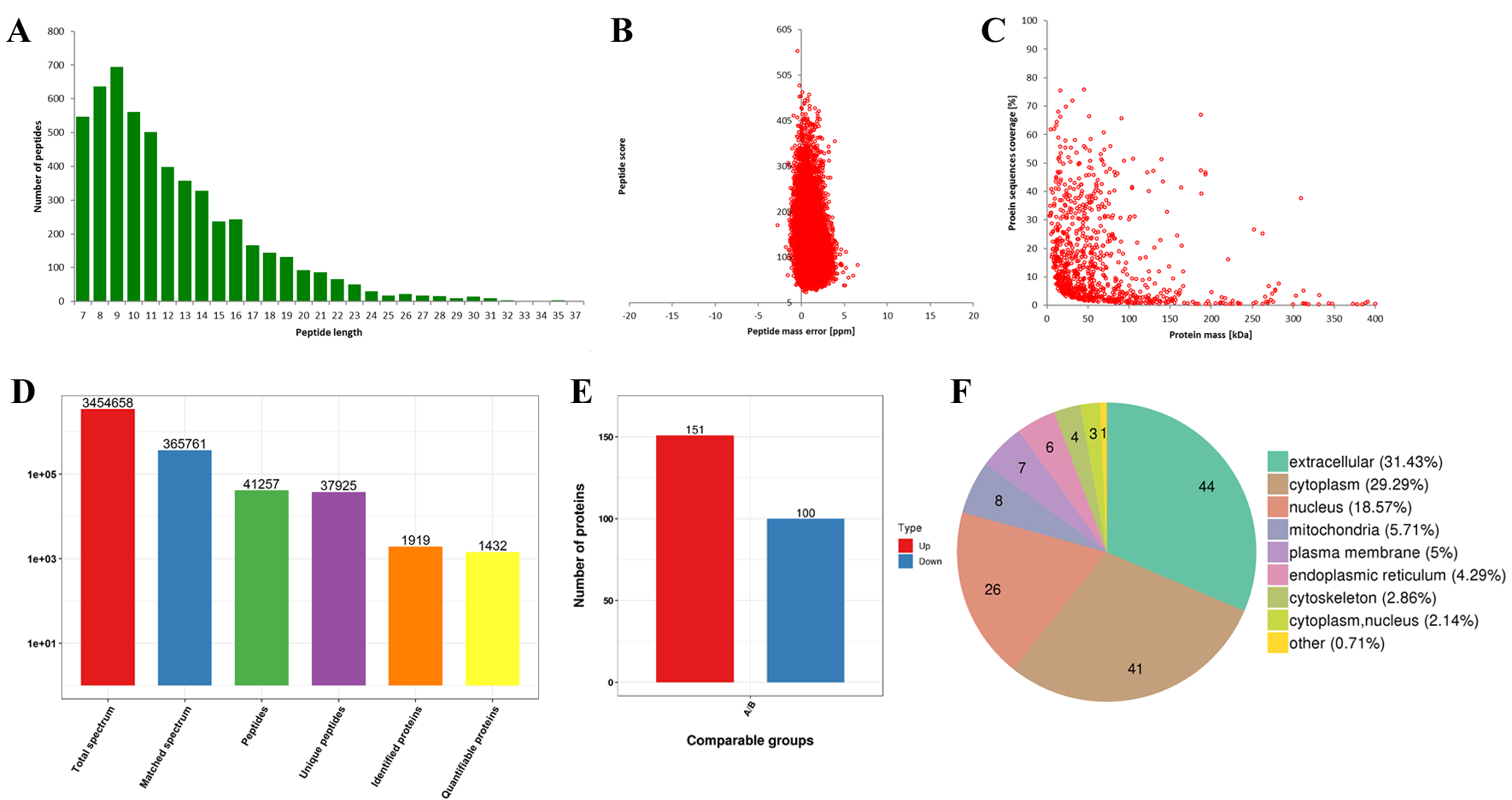

Supplement: Supplementary file 1 — Additional file 1: Figure S1. Quality control analysis of proteomic data. A. The lengths of peptides. B. The mass errors of peptides. C. Protein mass. D. Coverage and sequence distribution of the proteins. E. Number of proteins in comparable groups. F. Protein subcellular distribution. [file 12931_2023_2349_MOESM1_ESM.tif]

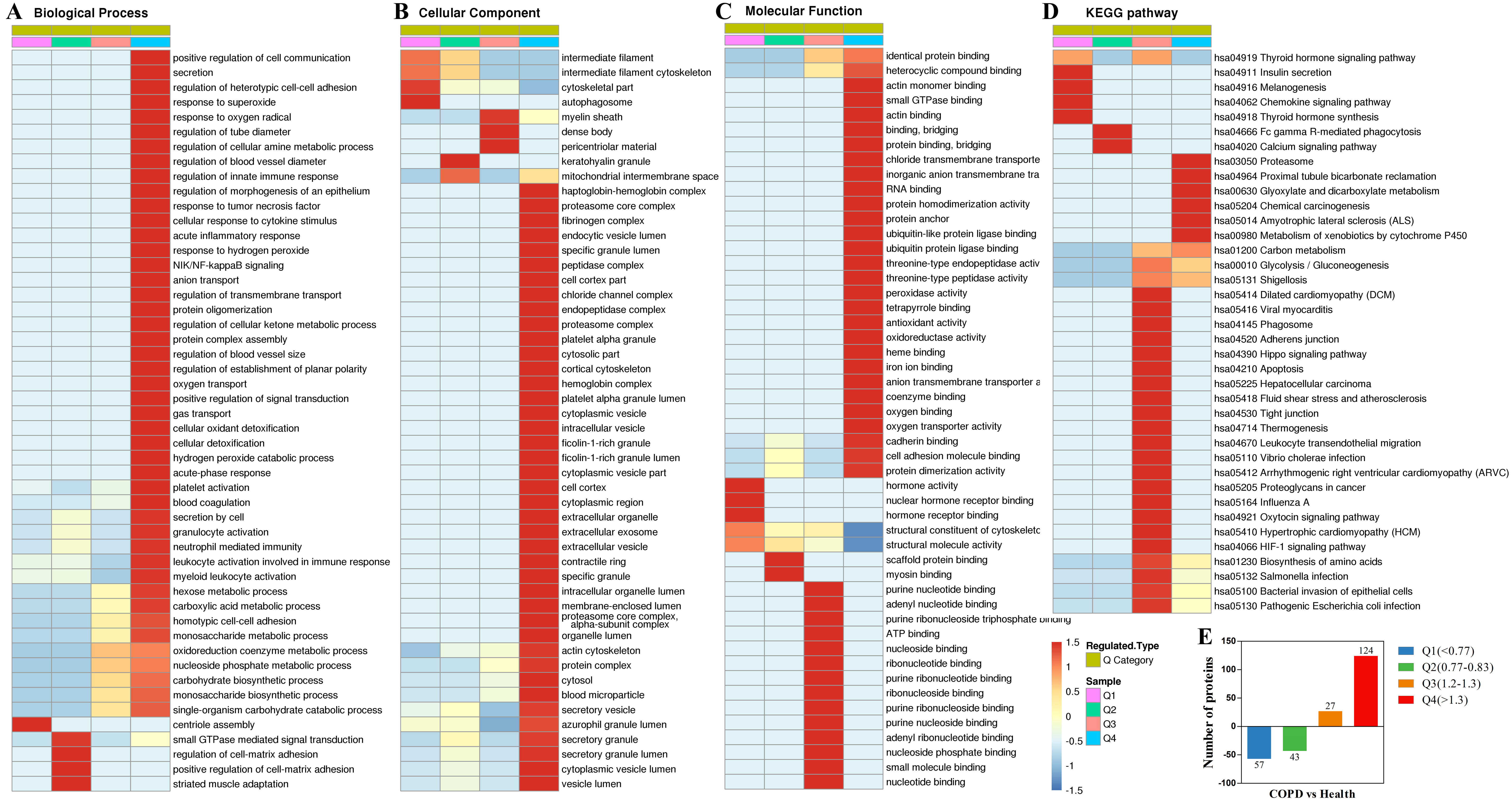

Supplement: Supplementary file 2 — Additional file 2: Figure S2. Heatmap showing of the DEPs analysis on GO and KEGG. The DEPs were divided into Q1–Q4 according to the multiple of fold change, and the heatmap of enrichment analysis preformed on GO and KEGG. [file 12931_2023_2349_MOESM2_ESM.tif]

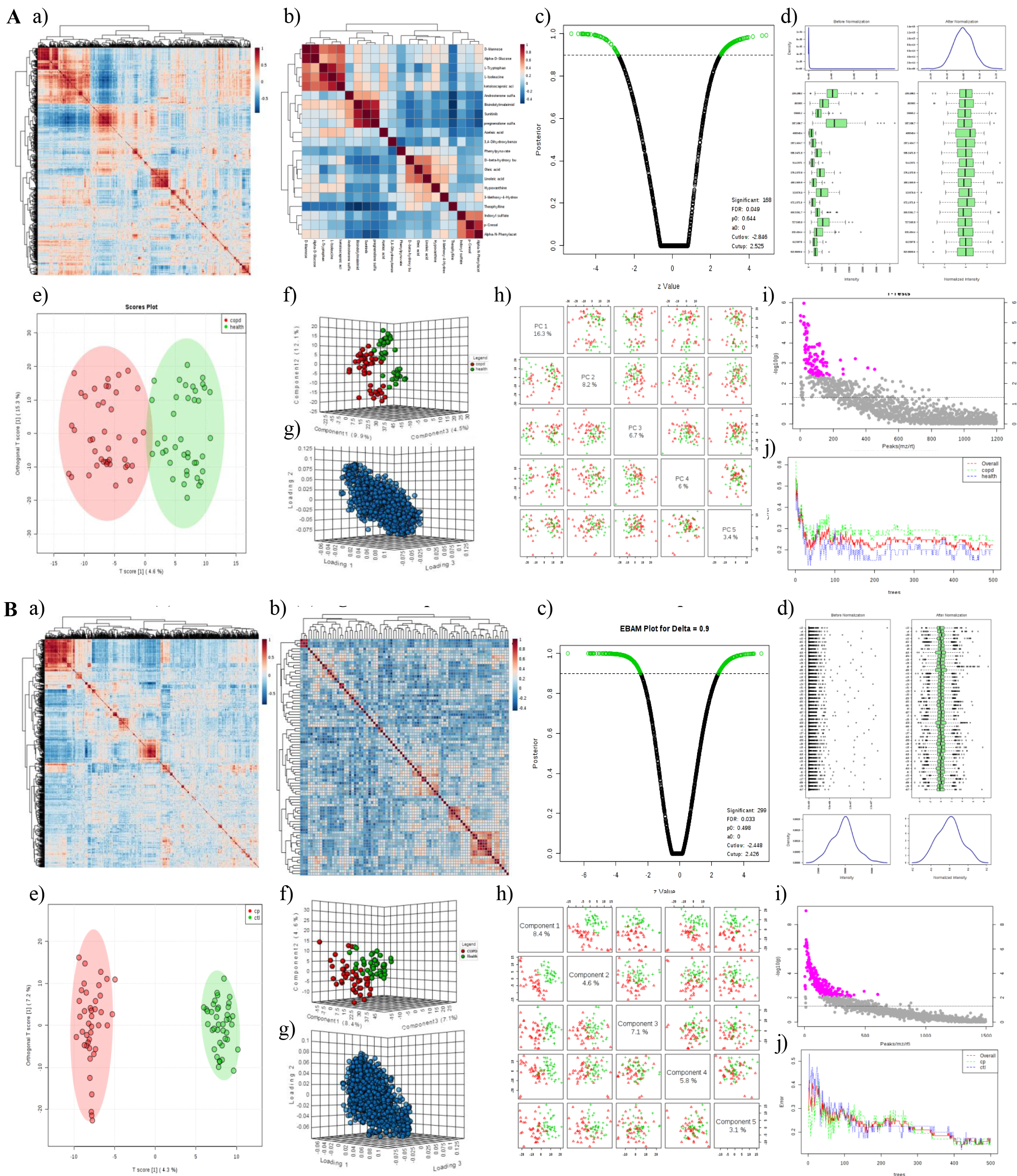

Supplement: Supplementary file 3 — Additional file 3: Figure S3. Quality control analysis of metabolomic data. Correlation distributions for positive and negative metabolites, respectively, and EBAM plots, normalization, PLS-DA, and t test generated. [file 12931_2023_2349_MOESM3_ESM.tif]

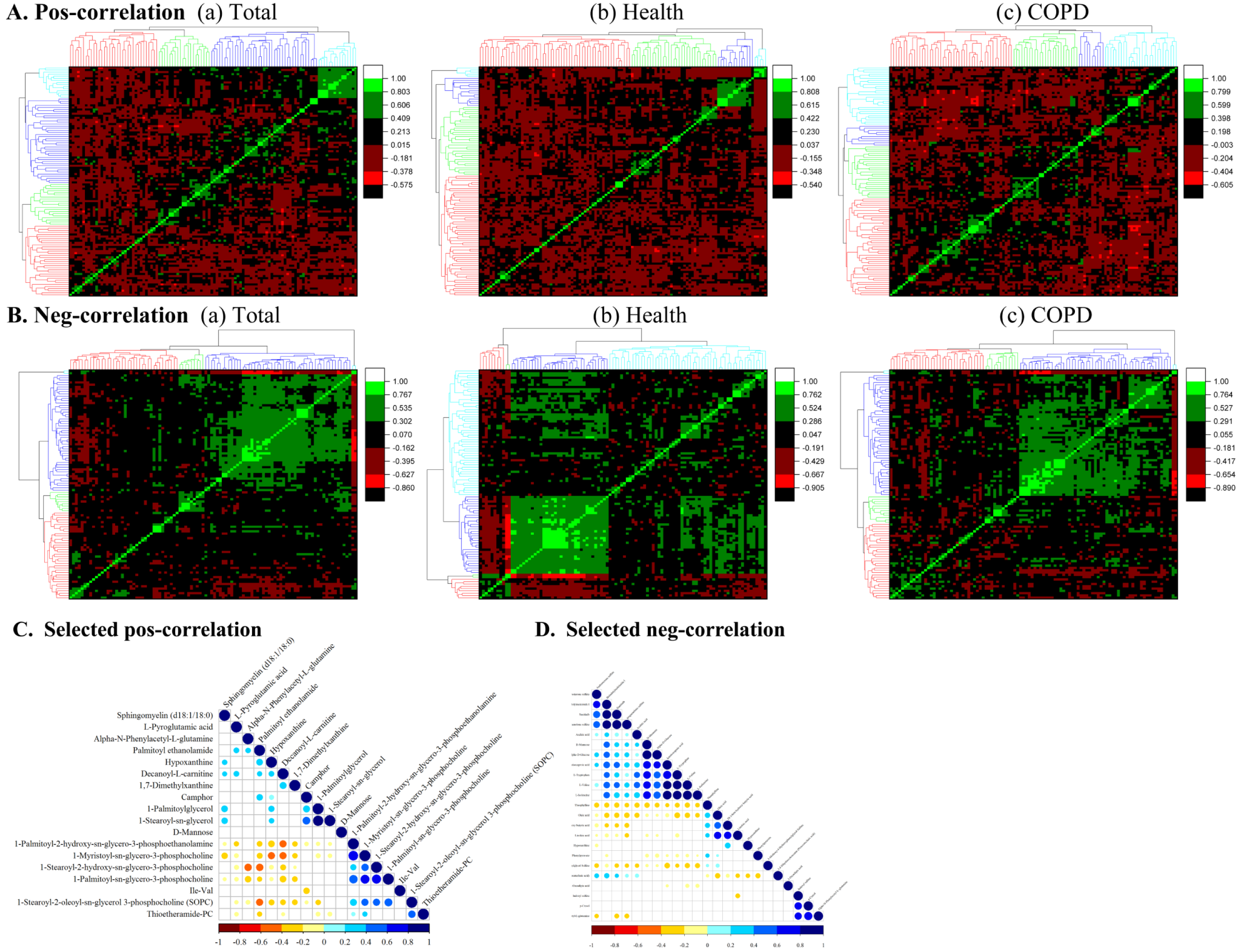

Supplement: Supplementary file 4 — Additional file 4: Figure S4. Correlation distributions for total and selected-metabolites. Correlation distributions for total and selected-metabolites grouped by COPD and healthy controls. [file 12931_2023_2349_MOESM4_ESM.tif]

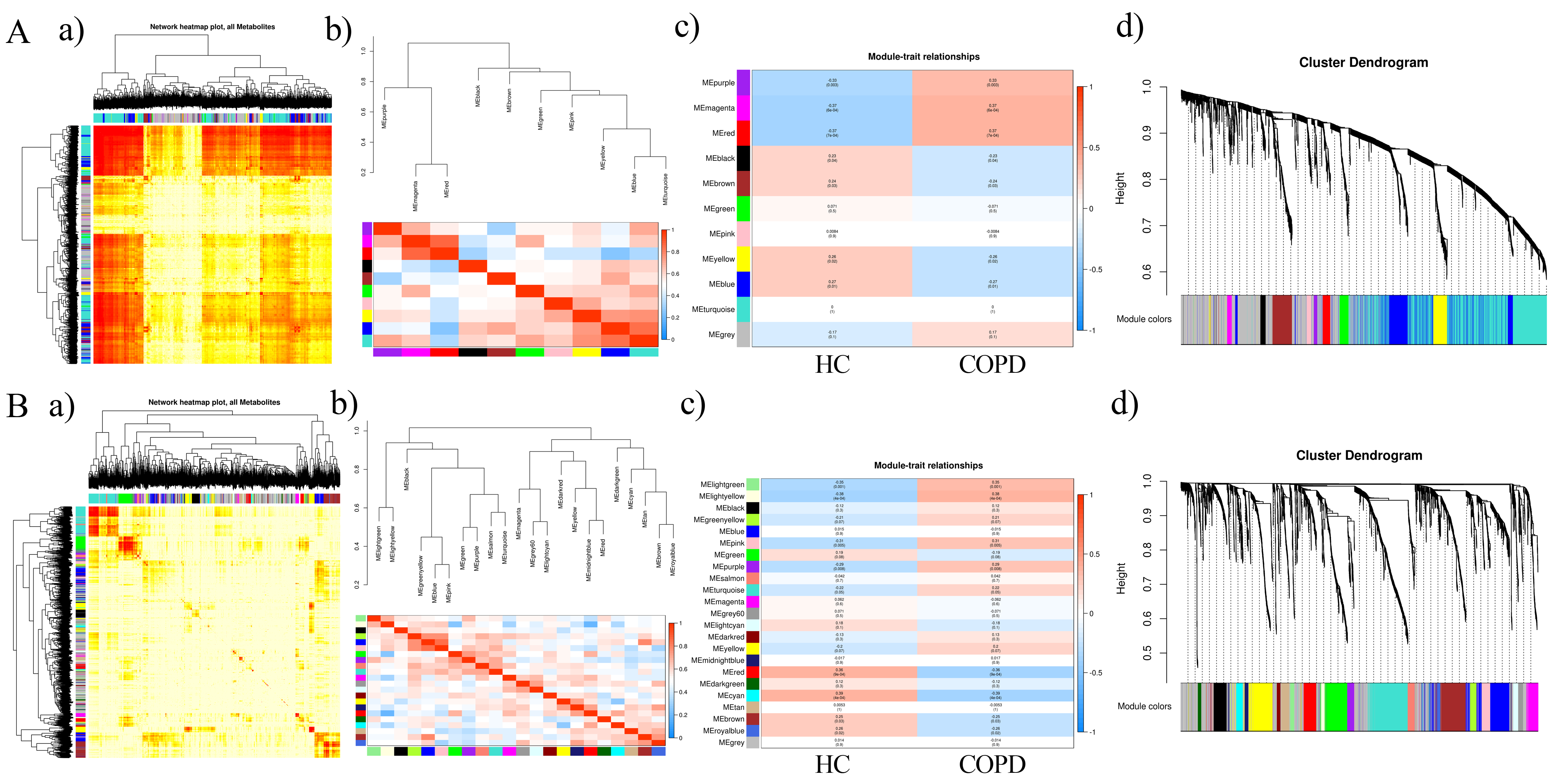

Supplement: Supplementary file 5 — Additional file 5: Figure S5. Metabolomics data analysis on the weighted gene co-expression network analysis (WGCNA) in COPD compared to healthy controls. A, Positive metabolites; B, Negative metabolites. [file 12931_2023_2349_MOESM5_ESM.tif]
