# Supplementary material for: Proteomics and metabolomics profiling reveal panels of circulating diagnostic biomarkers and molecular subtypes in stable COPD
Source: Respir Res. 2023 Mar 11;24:73. doi: 10.1186/s12931-023-02349-x (PMC10007826; doi:10.1186/s12931-023-02349-x)
Supplement: Supplementary file 6 — Additional file 6: Table S1. Proteins was generated for the targeted proteomic survey. [file 12931_2023_2349_MOESM6_ESM.docx]

Table S1. Proteins was generated for the targeted proteomic survey.

| **Protein accession** | **Gene** | **Regulated Type** | **Subcellular localization** | **KEGG KO No.** | **COPD/Control Ratio** | **P value** |
| --- | --- | --- | --- | --- | --- | --- |
| P02763 | ORM1 | Up | extracellular | K17308 | 2.74 | 1.36E-04 |
| P00738 | HP | Up | extracellular | K16142 | 2.27 | 3.76E-03 |
| P68871 | HBB | Up | cytoplasm | K13823 | 2.90 | 7.01E-04 |
| P18206 | VCL | Up | cytoplasm | K05700 | 1.37 | 1.30E-03 |
| P60174 | TPI1 | Up | cytoplasm | K01803 | 1.66 | 7.61E-05 |
| P69905 | HBA1 | Up | cytoplasm | K13822 | 3.58 | 1.82E-03 |
| P00918 | CA2 | Up | cytoplasm | K18245 | 2.03 | 4.86E-06 |
| P00441 | SOD1 | Up | cytoplasm | K04565 | 1.54 | 2.11E-05 |
| P02671 | FGA | Up | extracellular | K03903 | 1.74 | 1.36E-02 |
| P32119 | PRDX2 | Up | cytoplasm | K03386 | 2.10 | 4.37E-05 |
| P33151 | CDH5 | Up | plasma membrane | K06533 | 1.41 | 2.71E-10 |
| P04075 | ALDOA | Up | cytoplasm | K01623 | 1.49 | 4.70E-03 |
| P00915 | CA1 | Up | cytoplasm | K01672 | 2.23 | 1.50E-06 |
| J3QSU6 | TNC | Up | — | — | 1.41 | 2.96E-03 |
| P04040 | CAT | Up | cytoplasm | K03781 | 1.38 | 7.98E-04 |
| P02750 | LRG1 | Up | extracellular | — | 1.37 | 5.70E-05 |
