# Supplementary material for: Proteomics and metabolomics profiling reveal panels of circulating diagnostic biomarkers and molecular subtypes in stable COPD
Source: Respir Res. 2023 Mar 11;24:73. doi: 10.1186/s12931-023-02349-x (PMC10007826; doi:10.1186/s12931-023-02349-x)
Supplement: Supplementary file 7 — Additional file 7: Table S2. Predictive efficacy of single biomarker. [file 12931_2023_2349_MOESM7_ESM.docx]

Table S2. Predictive efficacy of single biomarker.

| Biomarkers | ROC analysis | | |  | Logistic regression | |
| --- | --- | --- | --- | --- | --- | --- |
|  | auROC | Sen. | Spe. |  | Coef. | P-value |
| Positive metabolites |  |  |  |  |  |  |
| Palmitoyl ethanolamide | 0.78 | 0.68 | 0.72 |  | 2.8e-5 | <0.001 |
| Decanoyl-L-carnitine | 0.73 | 0.56 | 0.68 |  | 8.9e-5 | 0.002 |
| trans-Dehydroandrosterone | 0.74 | 0.73 | 0.62 |  | 2.4e-4 | 0.001 |
| Betaine | 0.72 | 0.66 | 0.70 |  | 3.8e-4 | 0.003 |
| Pseudouridine | 0.76 | 0.68 | 0.70 |  | 2.4e-3 | <0.001 |
| 1-Stearoyl-2-hydroxy-sn-glycero-3-phosphocholine | 0.73 | 0.66 | 0.70 |  | 3.1e-6 | 0.001 |
| Camphor | 0.74 | 0.56 | 0.72 |  | 6.3e-5 | 0.001 |
| Hypoxanthine | 0.73 | 0.71 | 0.73 |  | 4.9e-5 | 0.001 |
| Negetive metabolites |  |  |  |  |  |  |
| 1-Stearoyl-sn-glycerol | 0.78 | 0.71 | 0.70 |  | 4.3e-4 | <0.001 |
| Theophylline | 0.74 | 0.54 | 0.88 |  | 5.9e-6 | 0.014 |
| Azelaic acid | 0.65 | 0.71 | 0.55 |  | 3.6e-5 | 0.013 |
| L-Isoleucine | 0.64 | 0.76 | 0.45 |  | 6.0e-6 | 0.018 |
| Sunitinib | 0.64 | 0.66 | 0.45 |  | 2.6e-6 | 0.029 |
| pregnenolone sulfate | 0.62 | 0.66 | 0.43 |  | 1.1e-5 | 0.029 |
| Bisindolylmaleimide I | 0.62 | 0.61 | 0.48 |  | 3.4e-5 | 0.058 |
| Androsterone sulfate | 0.62 | 0.71 | 0.43 |  | 7.6e-6 | 0.072 |
| Hypoxanthine | 0.72 | 0.51 | 0.72 |  | 6.4e-5 | 0.001 |
| Proteins |  |  |  |  |  |  |
| VCL | 0.71 | 0.57 | 0.78 |  | 5.74 | 0.002 |
| ORM1 | 0.72 | 0.54 | 0.92 |  | 2.10 | 0.007 |
| HBB | 0.72 | 0.46 | 0.80 |  | 1.03 | 0.023 |
| HP | 0.67 | 0.43 | 0.85 |  | 1.47 | 0.013 |
| CDH5 | 0.85 | 0.80 | 0.78 |  | 6.63 | <0.001 |
| PRDX2 | 0.75 | 0.55 | 0.90 |  | 1.97 | 0.002 |
| Blood routine test |  |  |  |  |  |  |
| Monocyte count | 0.64 | 0.59 | 0.53 |  | 2.97 | 0.037 |
| MPV | 0.68 | 0.62 | 0.63 |  | -0.65 | 0.026 |
