# Supplementary material for: Proteomics and metabolomics profiling reveal panels of circulating diagnostic biomarkers and molecular subtypes in stable COPD
Source: Respir Res. 2023 Mar 11;24:73. doi: 10.1186/s12931-023-02349-x (PMC10007826; doi:10.1186/s12931-023-02349-x)
Supplement: Supplementary file 8 — Additional file 8: Table S3. Predictive efficacy of the combined biomarkers. [file 12931_2023_2349_MOESM8_ESM.docx]

Table S3. Predictive efficacy of the combined biomarkers.

| Biomarkers | ROC analysis | | |  | Logistic regression | |
| --- | --- | --- | --- | --- | --- | --- |
|  | auROC | Sen. | Spe. |  | Coef. | P-value |
| Positive metabolites |  |  |  |  |  |  |
| Palmitoyl ethanolamide |  |  |  |  | 3.1e-5 | 0.001 |
| Decanoyl-L-carnitine |  |  |  |  | 9.8e-5 | 0.006 |
| trans-Dehydroandrosterone |  |  |  |  | 2.7e-4 | 0.009 |
| Betaine |  |  |  |  | 3.7e-4 | 0.019 |
| Summary of diagnosis performance of  P4 positive metabolites | 0.910 | 0.83 | 0.85 |  |  |  |
|  |  |  |  |  |  |  |
| Negetive metabolites |  |  |  |  |  |  |
| 1-Stearoyl-sn-glycerol |  |  |  |  | 4.4e-4 | 0.011 |
| Theophylline |  |  |  |  | 1.3e-5 | 0.037 |
| L-Isoleucine |  |  |  |  | 1.7e-5 | 0.007 |
| Hypoxanthine |  |  |  |  | 1.7e-4 | 0.001 |
| Summary of diagnosis performance of  P4 negetive metabolites | 0.959 | 0.90 | 0.90 |  |  |  |
|  |  |  |  |  |  |  |
| Proteins |  |  |  |  |  |  |
| ORM1 |  |  |  |  | 2.53 | 0.020 |
| CDH5 |  |  |  |  | 6.64 | <0.001 |
| PRDX2 |  |  |  |  | 1.94 | 0.033 |
| Summary of diagnosis performance of P3 proteins | 0.936 | 0.88 | 0.90 |  |  |  |
