# Supplementary material for: Proteomics and metabolomics profiling reveal panels of circulating diagnostic biomarkers and molecular subtypes in stable COPD
Source: Respir Res. 2023 Mar 11;24:73. doi: 10.1186/s12931-023-02349-x (PMC10007826; doi:10.1186/s12931-023-02349-x)
Supplement: Supplementary file 9 — Additional file 9: Table S4. Predictive efficacy of the combined biomarkers for the best serum biomarkers. [file 12931_2023_2349_MOESM9_ESM.docx]

Table S4. Predictive efficacy of the combined biomarkers for the best serum biomarkers.

| Biomarkers | ROC analysis | | |  | Logistic regression | |
| --- | --- | --- | --- | --- | --- | --- |
|  | auROC | Sen. | Spe. |  | Coef. | P-value |
| Metabolites |  |  |  |  |  |  |
| Palmitoyl ethanolamide |  |  |  |  | 4.1e-5 | 0.003 |
| Decanoyl-L-carnitine |  |  |  |  | 1.4e-4 | 0.006 |
| Betaine |  |  |  |  | 4.8e-4 | 0.021 |
| Theophylline |  |  |  |  | 1.5e-5 | 0.038 |
| Hypoxanthine |  |  |  |  | 1.2e-4 | 0.009 |
| Summary of diagnosis performance  of P5 metabolites | 0.970 | 0.88 | 0.93 |  |  |  |
|  |  |  |  |  |  |  |
| Combined biomarkers |  |  |  |  |  |  |
| Palmitoyl ethanolamide |  |  |  |  | 4.6e-5 | 0.014 |
| Theophylline |  |  |  |  | 1.3e-5 | 0.052 |
| Hypoxanthine |  |  |  |  | 7.7e-5 | 0.058 |
| CDH5 |  |  |  |  | 8.34 | 0.002 |
| Summary of diagnosis performance  of P4 combined biomarkers | 0.98 | 0.94 | 0.95 |  |  |  |
